# Analysis of the components of *Mycobacterium tuberculosis* heat-resistant antigen (Mtb-HAg) and its regulation of γδ T-cell function

**DOI:** 10.1186/s11658-024-00585-7

**Published:** 2024-05-13

**Authors:** Jing Wei, Fangzheng Guo, Yamin Song, Tong Feng, Ying Wang, Kun Xu, Jianhan Song, Eldana Kaysar, Reyima Abdukayyum, Feiyang Lin, Kangsheng Li, Baiqing Li, Zhongqing Qian, Xiaojing Wang, Hongtao Wang, Tao Xu

**Affiliations:** 1 Laboratory Medicine Experimental Center, Laboratory Medicine College, Bengbu Medical University, Bengbu, 233000 China; 2 Xinjiang Key Laboratory of Hotan Characteristic Chinese Traditional Medicine Research, College of Xinjiang Uyghur Medicine, Hotan, 848099, China; 3 Anhui Province Key Laboratory of Immunology in Chronic Diseases, Bengbu Medical University, Bengbu, 233000 China; 4 Anhui Province Key Laboratory of Clinical and Preclinical Research in Respiratory Disease, Bengbu Medical University, Bengbu, 233000 China

**Keywords:** *Mycobacterium tuberculosis* heat resistant antigen (Mtb-HAg), γδ T cell, TNF-α, IFN-γ

## Abstract

**Background:**

*Mycobacterium tuberculosis* heat-resistant antigen (Mtb-HAg) is a peptide antigen released from the mycobacterial cytoplasm into the supernatant of *Mycobacterium tuberculosis* (Mtb) attenuated H37Ra strain after autoclaving at 121 °C for 20 min. Mtb-HAg can specifically induce γδ T-cell proliferation in vitro. However, the exact composition of Mtb-HAg and the protein antigens that are responsible for its function are currently unknown.

**Methods:**

Mtb-HAg extracted from the Mtb H37Ra strain was subjected to LC‒MS mass spectrometry. Twelve of the identified protein fractions were recombinantly expressed in *Escherichia coli* by genetic engineering technology using pET-28a as a plasmid and purified by Ni–NTA agarose resin to stimulate peripheral blood mononuclear cells (PBMCs) from different healthy individuals. The proliferation of γδ T cells and major γδ T-cell subset types as well as the production of TNF-α and IFN-γ were determined by flow cytometry. Their proliferating γδ T cells were isolated and purified using MACS separation columns, and Mtb H37Ra-infected THP-1 was co-cultured with isolated and purified γδ T cells to quantify *Mycobacterium* viability by counting CFUs.

**Results:**

In this study, Mtb-HAg from the attenuated Mtb H37Ra strain was analysed by LC‒MS mass spectrometry, and a total of 564 proteins were identified. Analysis of the identified protein fractions revealed that the major protein components included heat shock proteins and Mtb-specific antigenic proteins. Recombinant expression of 10 of these proteins in by* Escherichia coli* genetic engineering technology was used to successfully stimulate PBMCs from different healthy individuals, but 2 of the proteins, EsxJ and EsxA, were not expressed. Flow cytometry results showed that, compared with the IL-2 control, HspX, GroEL1, and GroES specifically induced γδ T-cell expansion, with Vγ2δ2 T cells as the main subset, and the secretion of the antimicrobial cytokines TNF-α and IFN-γ. In contrast, HtpG, DnaK, GroEL2, HbhA, Mpt63, EsxB, and EsxN were unable to promote γδ T-cell proliferation and the secretion of TNF-α and IFN-γ. None of the above recombinant proteins were able to induce the secretion of TNF-α and IFN-γ by αβ T cells. In addition, TNF-α, IFN-γ-producing γδ T cells inhibit the growth of intracellular Mtb.

**Conclusion:**

Activated γδ T cells induced by Mtb-HAg components HspX, GroES, GroEL1 to produce TNF-α, IFN-γ modulate macrophages to inhibit intracellular Mtb growth. These data lay the foundation for subsequent studies on the mechanism by which Mtb-HAg induces γδ T-cell proliferation in vitro, as well as the development of preventive and therapeutic vaccines and rapid diagnostic reagents.

**Supplementary Information:**

The online version contains supplementary material available at 10.1186/s11658-024-00585-7.

## Background

Tuberculosis (TB), an infectious disease caused by *Mycobacterium tuberculosis* (Mtb), is one of the world's deadliest infectious diseases, with an estimated 1.6 million deaths in 2021 (including 187,000 people living with HIV) [1]. The spread of multidrug-resistant TB (MDR-TB), extensively drug-resistant TB (XDR-TB), and human immunodeficiency virus (HIV) and TB coinfection poses a major challenge to the global target of ending the TB epidemic by 2035 [2]. Therefore, an in-depth study of the pathogenic mechanism of TB is of great importance for the prevention and control of TB in the future.

γδ T cells, as an unconventional subset of T cells, have become increasingly popular in the field of immunotherapy in recent years. In healthy adults, γδ T cells account for only 1–5% of the total number of circulating lymphocytes, and their T cell receptor (TCR) consists of γ and δ chains that lack the diversity to directly recognize certain intact peptide antigens. Their activation does not require binding to major histocompatibility complex (MHC) or antigen presenting cell (APC) presentation [3, 4]. The Vγ2Vδ2 T-cell subset accounts for 65–90% of total circulating human γδ T cells and remains the only γδ T lymphocyte subset capable of recognizing phosphoantigens such as isopentenyl pyrophosphate metabolites produced by Mtb and other microorganisms and (E)-4-hydroxy-3-methyl-but-2-enyl pyrophosphate (HMBPP) [5, 6]. The main antigens recognized by γδ T cells identified thus far include MHC and MHC-like molecules, heat shock proteins (HSPs), DNA mismatch repair-associated proteins (MSH2), phosphorylated antigens, and lipid antigens, such as CD1a, CD1c, and CD1d, from the CD1 family [7]. IPP- or HMBPP-induced γδ T cells can readily produce the anti-tuberculosis cytokines IFN-γ and TNF-α, which are capable of inducing macrophage or monocyte activation and play important roles in the body's antitumour, anti-infection, immunomodulation, immunosurveillance, and maintenance of immune tolerance abilities [8–11]. Although CD4^+^ and CD8^+^ αβ T cells have been clearly demonstrated to play an important role in protective immunity against Mtb infection [12, 13], several lines of evidence over the past few years [14–16], including previous work from our laboratory [17, 18], suggest that γδ T cells may also play an important role in host immunity against Mtb infection.

Mtb heat-resistant antigen (Mtb-HAg) is an antigen released from the cytoplasm of the Mtb H37Ra strain after heat treatment at 121 ℃ that can specifically stimulate the proliferation of γδT cells in vitro [19, 20]. Mtb-HAg can also distinguish TB and latent tuberculosis infection (LTBI) by stimulating the production of different TNF-α and IFN-γ levels in human peripheral blood T cells [21, 22]. Wang et al. demonstrated that the proliferation of NK cells, γδNKT cells and γδT cells could be induced after stimulation with Mtb-HAg. γδNKT cells and γδT cells proliferate, while the number of NK cells decreases after 11 days of simulation with Mtb-HAg [23]. Zhang et al. [24] found that Mtb-HAg can specifically activate NK cells, and activated NK cells participate in regulating the response of γδT cells to Mtb-HAg by secreting cytokines (GM-CSF, TNF-α, IL-22) and intercellular contact. However, the composition of Mtb-HAg and the protein antigens that perform its function are currently unknown. If specific protein antigens can be screened and identified from Mtb-HAg and used to develop TB preventive and therapeutic vaccines as well as specific diagnostic reagents, it will enhance the rapid diagnosis and treatment of TB.

In the present study, Mtb-HAg was prepared by heating the Mtb H37Ra strain at 121 °C for 20 min, and its specific stimulation of γδT cell proliferation was verified in vitro*.* Then, liquid chromatography coupled to mass spectrometry (LC‒MS) was used to separate and characterize the digested Mtb-HAg, and the characteristic fragments of the Mtb-HAg protein were screened out. Finally, we identified the effect of the Mtb-HAg protein on the function of γδ T cells. Altogether, our data demonstrated that Mtb-HAg proteins (HspX, GroEL1 and GroES) specifically induce the expansion of γδ T cells, with Vγ2δ2 T cells as the main subset, and promote the secretion of TNF-α and IFN-γ. Activated γδ T cells induced by Mtb-HAg components HspX, GroES, GroEL1 to produce TNF-α, IFN-γ modulate macrophages to inhibit intracellular Mtb growth.

## Materials and methods

### Preparation of Mtb-HAg

Mtb-HAg was prepared according to previous reports [18, 19, 24–26]. Briefly, Mtb H37Ra was cultured in Middlebrook 7H11 agar medium (Thermo Fisher Scientific, USA) and incubated at 37 °C for 21 days. They were then cultured in 300 mL Sauton’s medium (l-Asparagine 4.0 g, Citric acid 2.0 g, Dipotassium hydrogen phosphate 0.5 g, Anhydrous magnesium sulfate 0.24 g, Ammonium iron citrate 0.05 g, 60 mL glycerolin in 940 mL ultra pure water, adjusting pH to 7.0, Solarbio, Beijing, China) for 4–5 weeks until they reached logarithmic growth phase (Once the stationary phase is reached, Mtb will age and die, which is not conducive to subsequent research). The mycobacterial cells were harvested, washed three times with normal saline (0.9% NaCl solution), centrifuged for 30 min at 4000*g*. The volume of ultrapure water added is 2 times the volume of cell pellets after centrifugation, followed by heating at 121 °C for 20 min. Soluble Mtb-HAg was collected from the supernatant of heat-treated Mtb cells. The supernatant was removed and filtered (0.22 μm-pore-size filter), adjusted in H_2_O at a concentration of 1.0 mg/mL using BCA Protein Assay Kit (Beyotime, Shanghai, China), and stored at 4 ℃ for later use.

### Identification and analysis of Mtb-HAg by LC–MS mass spectrometry

Mtb-HAg digest peptide sequences were detected by LC–MS using a Q Exactive mass spectrometer (Thermo Fisher Scientific, MA, USA) and Dionex Ultimate 3000 RSLCnano liquid chromatography (Thermo Fisher Scientific, MA, USA). The injection volume is 6 μL in LC–MS analysis. Column information: 300 µm idx 5 mm, Acclaim PepMap RSLC C18, 5 µm, 100 A (Thermo, 160,454); Acclaim PepMap 75 µm × 150 mm, C18, 3 µm, 100 A (Thermo Fisher Scientific, MA, USA). The elution conditions for LC were as follows: eluent A consisted of 0.1% formic acid in Milli-Q water, eluent B consisted of 0.1% formic acid and 80% acetonitrile in Milli-Q water, and the flow rate was set to 300 nL/min. The elution procedure was as follows: 0–5 min, 5% B; 5–45 min, 50% B; 45–55 min, 90% B; 55–65 min, 5% B. The MS parameters for Mtb-HAg analysis were set as follows: resolution = 70 k; scan range (m/z) = 350–1800; maximum injection time = 40 ms; AGC target = 300, 0000. The MS/MS parameters for Mtb-HAg analysis were set as follows: resolution = 17.5 k; AGC target = 100,000; maximum injection time = 60 ms; NCE/stepped NCE = 27. The mass spectrometry raw files were processed and converted by Proteome Discoverer 1.4 software to obtain MGF format files, and then the uniprot database (Mtb H37Ra (https://www.uniprot.org/taxonomy/419947) was searched using MASCOT (http://www.matrixscience.com/).

### Expression and purification of Mtb-HAg components

Gene sequences of H37Ra strains HtpG (MRA_2316), DnaK (MRA_0359), GroEL2 (MRA_0445), GroEL1 (MRA_3457), HspX (MRA_2046), GroES (MRA_3458), HbhA (MRA_0482), Mpt63 (MRA_1937), EsxB (MRA_3913), EsxJ (MRA_1046), EsxA (MRA_3914) and EsxN (MRA_1806) were obtained from UniProt (https://www.uniprot.org/UniProt/). Using Mtb H37Ra genomic DNA as a template, the target gene was obtained by PCR amplification, and the primers are shown in Additional file 1: Table S1. We inserted the target gene into the vector pET-28a using the homologous recombination cloning technique [27]. The obtained 12 recombinant plasmids were confirmed by colony PCR and DNA sequencing.

The identified recombinant plasmids were separately transformed into *Escherichia coli* BL21 (DE3) competent cells for the induction of expression and incubated at 37 °C until an optical density of 0.6 at 600 nm was reached. Afterwards, 0.5 mM Isopropyl β-d-1-thiogalactopyranoside (IPTG) was added, and the culture was allowed to grow for 4 h for induction of recombinant protein expression. The cells were harvested by centrifugation, resuspended in lysis buffer (20 mM Tris buffer, 150 mM NaCl, and 1 mM PMSF, pH 8.0) containing 100 µg/mL lysozyme and sonicated in an ice bath. Finally, the supernatant and precipitate were collected separately by centrifugation at 10,000*g* for 10 min. The supernatant and precipitate were separated by SDS–PAGE.

His-tagged recombinant proteins were purified by nitrilotriacetic acid (Ni–NTA) agarose resin (CWBIO, Jiangsu, China) according to previous reports [28] for purification of proteins under soluble conditions. First, the crude supernatant cell extract was filtered through a 0.45 μm filter. Subsequently, the supernatant was loaded onto a Ni–NTA affinity column that had already been equilibrated with the lysis buffer and then washed with 50 mL of washing buffer (20 mM Tris–HCl, 150 mM NaCl, 10 mM imidazole, pH 8.0). Finally, the target protein was eluted with elution buffer (20 mM Tris–HCl, 150 mM NaCl, 500 mM imidazole, pH 8.0). The purified proteins were identified by SDS‒PAGE and Western blotting. For Western blotting, proteins separated by SDS–PAGE were transferred to PVDF membranes (Millipore, Bedford, USA) and immunoblotted with anti-His mouse monoclonal antibody (Beyotime, Shanghai, China). The secondary antibodies used were horseradish peroxidase-labelled goat anti-mouse IgG (H + L) (Beyotime, Shanghai, China). The purified protein concentrations were quantified using the BCA Protein Assay Kit (Beyotime, Shanghai, China).

### Study participants

Peripheral blood samples were obtained from students of the Bengbu Medical University (*n* = 8, aged 21 ± 2 years). All participants had similar ethnic backgrounds, no recent history of infectious diseases, and normal chest X-rays on physical examination. The exclusion criteria included cancer and autoimmune diseases. Peripheral blood samples from participants were collected after obtaining informed consent. Ethics approval for the present study was obtained by the Ethics Committee of Bengbu Medical College (approval no. BBMC-2022-68).

### Blood processing and in vitro stimulation

Peripheral blood mononuclear cells (PBMCs) were obtained from freshly heparinized venous blood from healthy adult peripheral blood by density gradient centrifugation using human lymphocyte isolation solution (TBD science, Tianjin, China). PBMCs (1 × 10^6^/mL) were seeded in 24-well culture plates (Labselect, Hefei, China) in complete RPMI-1640 medium (1 mL/well) (Gibco, USA), supplemented with 10% (v/v) heat-inactivated foetal bovine serum (FBS, EVERY GREEN, Hangzhou, China), 100 U/mL penicillin and 100 µg/mL streptomycin and cultured at 37 °C in 5% CO_2_. rhIL-2 (50 U/mL) (PeproTech, USA), HDMAPP (5 μg/mL), Mtb-HAg (5 μg/mL), GroES (5 μg/mL), GroEL1 (5 μg/mL), HspX (5 μg/mL), GroEL2 (5 μg/mL), HtpG (5 μg/mL), DnaK (5 μg/mL), HbhA (5 μg/mL), Mpt63 (5 μg/mL), EsxB (5 μg/mL), and EsxN (5 μg/mL) were added to each well, and rhIL-2 (50 U/mL) was added to each well every 3–4 days.

### Flow cytometry analysis and antibodies

PBMCs were washed twice in washing buffer (2% FBS-0.1% sodium azide in PBS) and then stained with fluorochrome-conjugated Abs. The following Abs were used for surface and intracellular cytokine staining for flow cytometry: CD3-APC (UCHT1, Biolegend, USA), **γ**δTCR-PE (B1, Biolegend, USA), Vδ2TCR-FITC (B6, Biolegend, USA), IFN-γ-PE/Cyanine7 (4S. B3, Biolegend, USA) and TNF-α-Brilliant Violet 421™ (MAb11, Biolegend, USA). All Ab incubations were conducted on ice for 30 min each. Next, the cells were washed three times with washing buffer, fixed in 2% paraformaldehyde (PFA, Biosharp, China), and then analysed by a DxP Athena instrument (Cytek, USA). The data were analysed with FlowJo software (Treestar, San Carlos, CA, USA). The expression of IFN-γ and TNF-α in PBMCs was determined by intracellular cytokine assays. Briefly, PBMCs were cultured for 9 days, restimulated with the same stimuli and concentrations as described above and incubated at 37 °C and 5% CO_2_ for 1 h, followed by an additional 5 h incubation in the presence of Monensin (1 µg/mL) (Biolegend, USA). Then, the cells were stained with CD3-APC and γδTCR-PE, Vδ2TCR-FITC for 30 min at 4 °C in the dark. After washing, fixation, and permeabilization, the cells were incubated with IFN-γ-PE/Cyanine7 and TNF-α-Brilliant Violet 421™ for 30 min at 4 °C in the dark. The concentration of the antibodies used above were 5 µL per million cells in 100 µL staining volume. After staining, cells were subjected to FCM (DxP Athena) and the data was analysed with FlowJo software.

### Determination of bacterial culture, cellular infection and intracellular mycobacterial growth

H37Ra-infected THP-1 were prepared as target cells at MOI = 10 as reported in the literature for 4 h [29–31]. The extracellular uninternalized bacilli were then removed by washing with pre-warmed PBS. To isolate or purify γδ T cells, γδ T cells were then purified using MACS separation columns (Miltenyi Biotech, German) according to the manufacturer's protocols, and they were used as effector cells. Flow cytometry was performed to assess the purity of purified γδ T cells induced by different stimulants. Target cells (5 × 10^4^ cells/well) alone were cultured with culture medium or purified effector cells (5 × 10^5^ cells/well) in 96-well plates at a ratio of E:T = 10:1 for 3 d. Wells were then aspirated and infected cells were lysed with 200 µL of sterile PBS containing 0.067% SDS. A tenfold dilution was performed for quantitative incubation. 100 µL suspension was placed in antibiotic-free Mtb solid medium and left for 2–3 weeks until colonies were large enough to be counted. Mycobacterial viability was quantified by counting CFUs.

### Statistical analysis

Data are presented as means ± standard deviations. The data were analysed by one-way ANOVA followed by Dunnett’s multiple comparisons test of multiple comparisons to determine whether there were significant differences between individual groups. Statistical analysis was performed with GraphPad Prism 9.0 (San Diego, CA, USA). *P*-values < 0.05 were considered significant.

## Results

### LC–MS mass spectrometry analysis of Mtb-HAg components

**Fig. 1:**
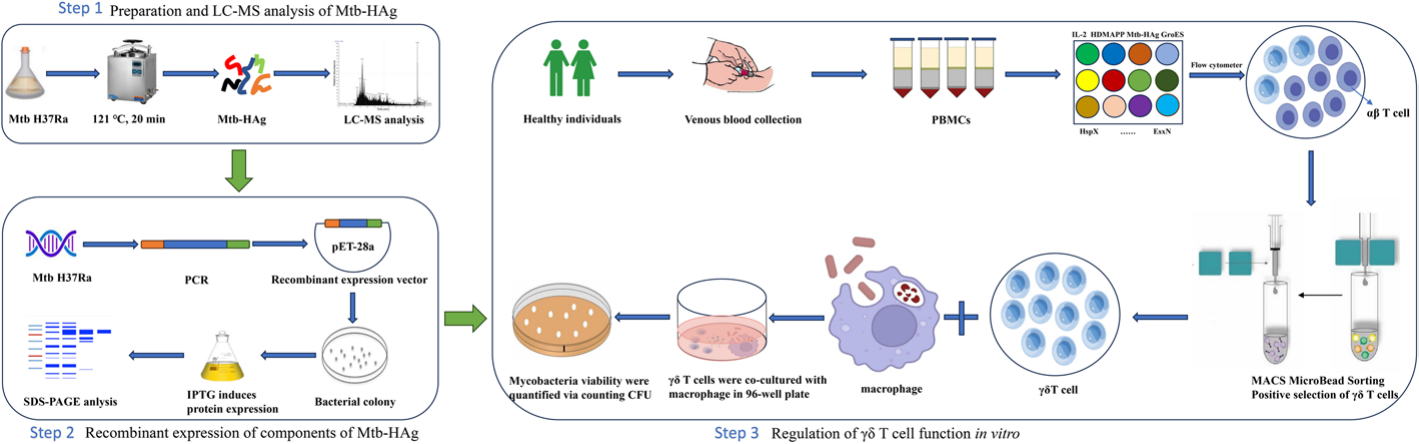
Workflow for analysing the components of Mtb-HAg and their regulation of γδ T-cell function

A workflow for the composition analysis of Mtb-HAg and their regulation of γδ T-cell function is shown in Fig. 1. To identify the main components of Mtb-HAg that selectively induce γδ T cell proliferation in human PBMCs in vitro, LC–MS mass spectrometry was used to characterize the Mtb-HAg components. Analysis of the protein data acquired by LC–MS mass spectrometry showed that a total of 625 protein fractions were obtained, and 564 proteins on pep_expect < 0.05 peptide matches were searched with MASCOT (http://www.matrixscience.com/) and screened (Additional file 2: Table S2). Several heat shock proteins were identified in Mtb H37Ra, such as GroEL1, GroEL2, HspX, DnaK, and HtpG, of which GroEL2, GroEL1, HspX, and DnaK ranked in the top 10. According to the results of mass spectrometry analysis, a total of 12 proteins were selected for subsequent analyses in this study by prot_score, prot_mass, prot_pi, prot_cover: GroES, GroEL1, HspX, GroEL2, HtpG, DnaK, HbhA, Mpt63, EsxB, EsxN, EsxJ, and EsxA (Table 1).

**Table 1:** Identification of Mtb attenuated strain H37Ra Mtb-HAg components by LC–MS mass spectrometry technique

Accession No.	Protein name	Score	prot_mass	PI	Coverage %
CH602_MYCTA	Chaperonin GroEL 2	4222	56,692	4.85	45.6
DNAK_MYCTA	Chaperone protein DnaK	3305	66,790	4.85	32.5
A5U456_MYCTA	Antigen Hsp20(HspX)	1909	16,217	5	35.4
CH601_MYCTA	Chaperonin GroEL 1	1269	55,843	4.98	31.4
CH10_MYCTA	Co-chaperonin GroES	902	10,798	4.62	55
HBHA_MYCTA	Heparin-binding hemagglutinin (HbhA)	520	21,511	9.17	40.7
HTPG_MYCTA	Chaperone protein HtpG	426	73,030	4.79	14.8
A5U9K2_MYCTA	ESAT-6-like protein EsxB	318	10,787	4.59	63
A5U3U5_MYCTA	Immunogenic protein Mpt63	117	16,504	4.92	23.9
A5U3G0_MYCTA	ESAT-6-like protein EsxN	102	9993	4.76	31.9
A5U9K3_MYCTA	ESAT-6-like protein EsxA	100	9898	4.48	20
A5U182_MYCTA	ESAT-6-like protein EsxJ	19	10,986	5.17	36.7

### Cloning, expression and purification of the Mtb-HAg proteins

**Fig. 2:**
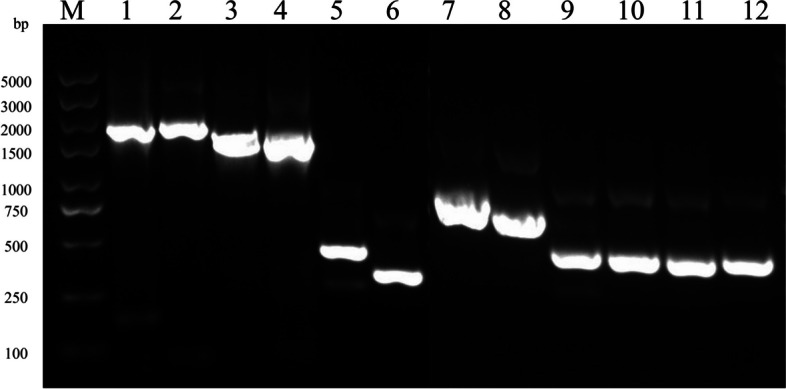
Electrophoresis analyses of PCR products. M: DNA Marker; 1: HtpG; 2: DnaK; 3: GroEL2; 4: GroEL1; 5: HspX; 6: GroES; 7: HbhA; 8: Mpt63; 9: EsxB; 10: EsxJ; 11: EsxA; 12: EsxN

Using Mtb H37Ra genomic DNA as a template, HtpG (1944 bp), DnaK (1878 bp), GroEL2 (1623 bp), GroEL1 (1620 bp), HspX (435 bp), GroES (303 bp), HbhA (600 bp), Mpt63 (480 bp), EsxB (303 bp), EsxJ (297 bp), EsxA (288 bp), and EsxN (285 bp) were obtained by PCR amplification (Fig. 2). The target genes were cloned and inserted into the pET28a vector through the homologous recombination cloning technique, and the positive clones obtained by colony PCR (Additional file 3: Fig. S1) and DNA sequencing (Additional file 4) were named pET28a-HtpG, pET28a-DnaK, pET28a-GroEL2, pET28a-GroEL1, pET28a-HspX, pET28a-GroES, pET28a-HbhA, pET28a-Mpt63, pET28a-EsxB, pET28a-EsxJ, pET28a-EsxA and pET28a-EsxN.

**Fig. 3:**
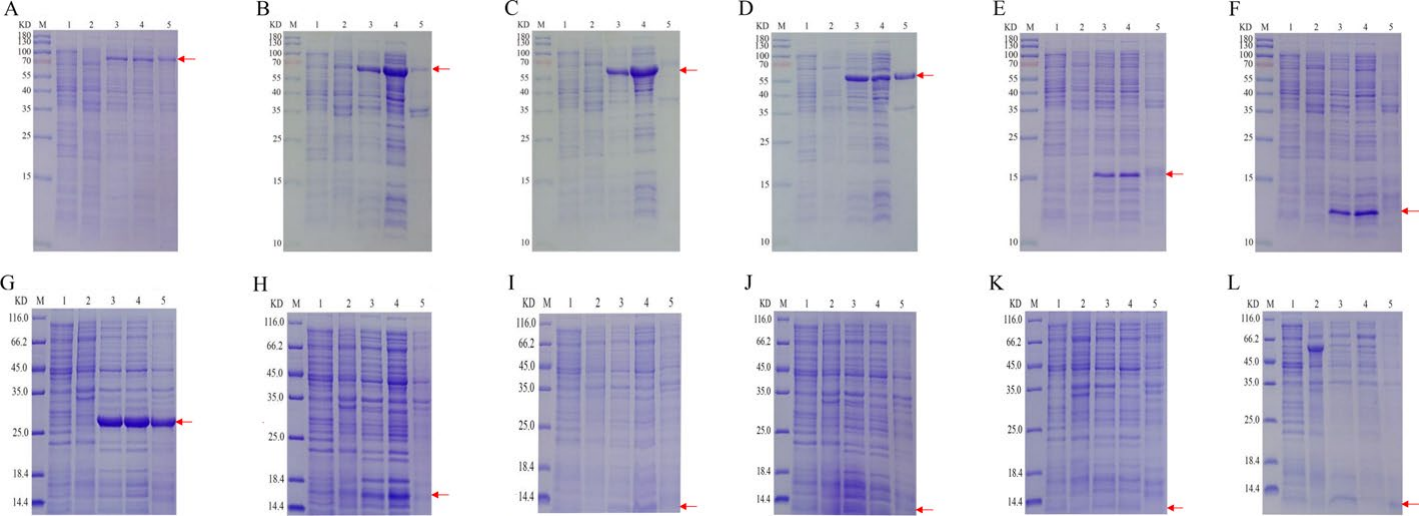
SDS‒PAGE analysis of the recombinant proteins expressed in *Escherichia coli*. M: Protein marker; 1: Escherichia coli BL21 without transformation; 2: Recombinant cells before induction with IPTG; 3: Recombinant cells after induction with IPTG; 4: Supernatant of lysate; 5: Precipitate of lysate. **A** HtpG; **B** DnaK; **C** GroEL2; **D** GroEL1; **E** HspX; **F** GroES; **G** HbhA; **H** Mpt63; **I** EsxB; **J** EsxJ; **K** EsxA; **L** EsxN

**Fig. 4:**
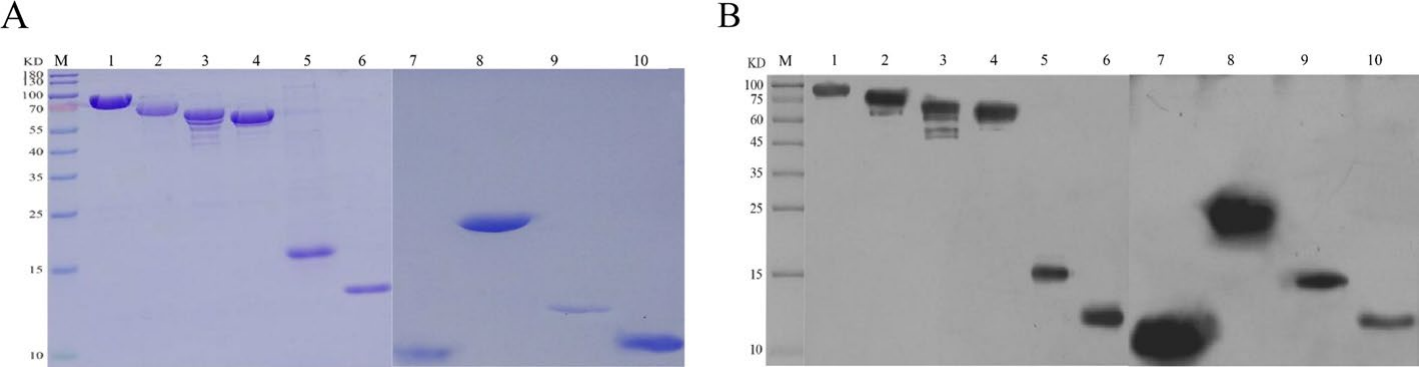
The purified recombinant proteins were analysed by SDS‒PAGE and Western blotting. **A** SDS‒PAGE analysis of the purified recombinant proteins. M: Protein marker; 1: HtpG; 2: DnaK; 3: GroEL2; 4: GroEL1; 5: HspX; 6: GroES; 7: EsxN; 8: HbhA; 9: Mpt63; 10: EsxB; **B** Western blot analysis of the purified recombinant proteins. M: Protein Marker; 1: HtpG; 2: DnaK; 3: GroEL2; 4: GroEL1; 5: HspX; 6: GroES; 7: EsxN; 8: HbhA; 9: Mpt63; 10: EsxB

The recombinant plasmids were transformed into the Escherichia coli expression strain BL21(DE3), and the induction of expression was achieved after incubation at 37 ℃ with 0.5 mM IPTG for 4 h. Afterwards, the total lysates were analysed by SDS-PAGE. As shown in Fig. 3A–L, we successfully expressed HtpG (75 kDa), DnaK (70 kDa), GroEL2 (60 kDa), GroEL1 (60 kDa), HspX (16 kDa), GroES (11 kDa), HbhA (27 kDa), Mpt63 (18 kDa), EsxB (11 kDa), and EsxN (11 kDa). Unfortunately, EsxJ (11 kDa) and EsxA (11 kDa) were not expressed (Fig. 3J–K). Then, the supernatant and precipitate were analysed by SDS-PAGE. The analysis revealed that DnaK (Fig. 3B), GroEL2 (Fig. 3C), HspX (Fig. 3E), GroES (Fig. 3F), Mpt63 (Fig. 3H), EsxB (Fig. 3I) are expressed in the supernatant (Fig. 3), while HtpG (Fig. 3A), GroEL1 (Fig. 3D), HbhA (Fig. 3G), EsxN (Fig. 3L) are expressed in the supernatant and precipitate (Fig. 3). The recombinant proteins were purified by a Ni–NTA affinity column to obtain greater than 90% purity (Additional file 5: Fig. S2A–J). Finally, the purified recombinant proteins were confirmed by SDS-PAGE (Fig. 4A) and Western blot analysis (Fig. 4B). Overall, we successfully expressed and purified HtpG, DnaK, GroEL2, GroEL1, HspX, GroES, HbhA, Mpt63, EsxB and EsxN.

### Recombinant proteins of Mtb-HAg components specifically induce the proliferation of human peripheral blood γδ T cells

**Fig. 5:**
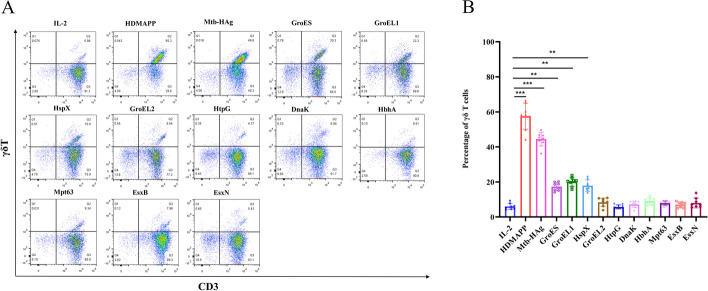
Analysis of Mtb-HAg and recombinant proteins stimulated peripheral blood γδ T-cell proliferation. **A** Flow chart of γδ T-cell proliferation. **B** Statistical analysis of γδ T-cell proliferation. (*P < 0.05, **P < 0.01, ***P < 0.001)

Although CD4^+^/CD8^+^ T cells have a protective role in human tuberculosis and other infections, the role of γδ T cells is unknown. Vγ2Vδ2 T cells, which are found only in primates, represent the major circulating γδ T-cell subset that recognizes phosphoantigens derived from Mtb and some specific pathogens. To determine whether the recombinant proteins induced γδ T-cell expansion in vitro, we stimulated PBMCs from different healthy individuals with the purified recombinant proteins and measured γδ T-cell proliferation at 9–12 days by flow cytometry. As shown in Fig. 5, GroES (17.35 ± 2.22%), GroEL1 (20.08 ± 2.42%), and HspX (17.91 ± 3.06%) stimulated the proliferation of γδ T cells in the peripheral blood of different healthy individuals up to approximately 20%, which was statistically higher than that of the negative control IL-2 group (6.02 ± 1.65%) by a factor of 3–5, and the activated γδ T cells were mainly dominated by Vγ2Vδ2 T cell subset (Additional file 6: Fig. S3). Vγ2Vδ2 T cells accounted for (88.49 ± 3.84%) of GroES-activated γδ T cells, (88.65 ± 6.06%) of GroEL1-activated γδ T cells, (86.36 ± 4.17%) of HspX-activated γδ T cells. The Vδ2 subset is the most important functional subset of γδ T cells in the peripheral blood. GroEL2 (8.43 ± 2.24%), HtpG (5.76 ± 1.10%), DnaK (7.18 ± 1.77%), HbhA (9.09 ± 1.80%), Mpt63 (7.94 ± 1.18%), EsxB (7.07 ± 1.46%), and EsxN (8.05 ± 2.67%) stimulated the proliferation of γδ T cells but did not induce a statistically significant increase compared to the negative IL-2 control group (6.02 ± 1.65%). Thus, these results suggest that the stimulation of PBMCs with GroES, GroEL1, and HspX induces a large expansion of γδ T cells with a predominance of the Vγ2Vδ2 T-cell subset.

### Mtb-HAg recombinant proteins directly induce TNF-α and IFN-γ production by human peripheral blood γδ T cells but do not induce TNF-α and IFN-γ production by human peripheral blood αβ T cells

**Fig. 6:**
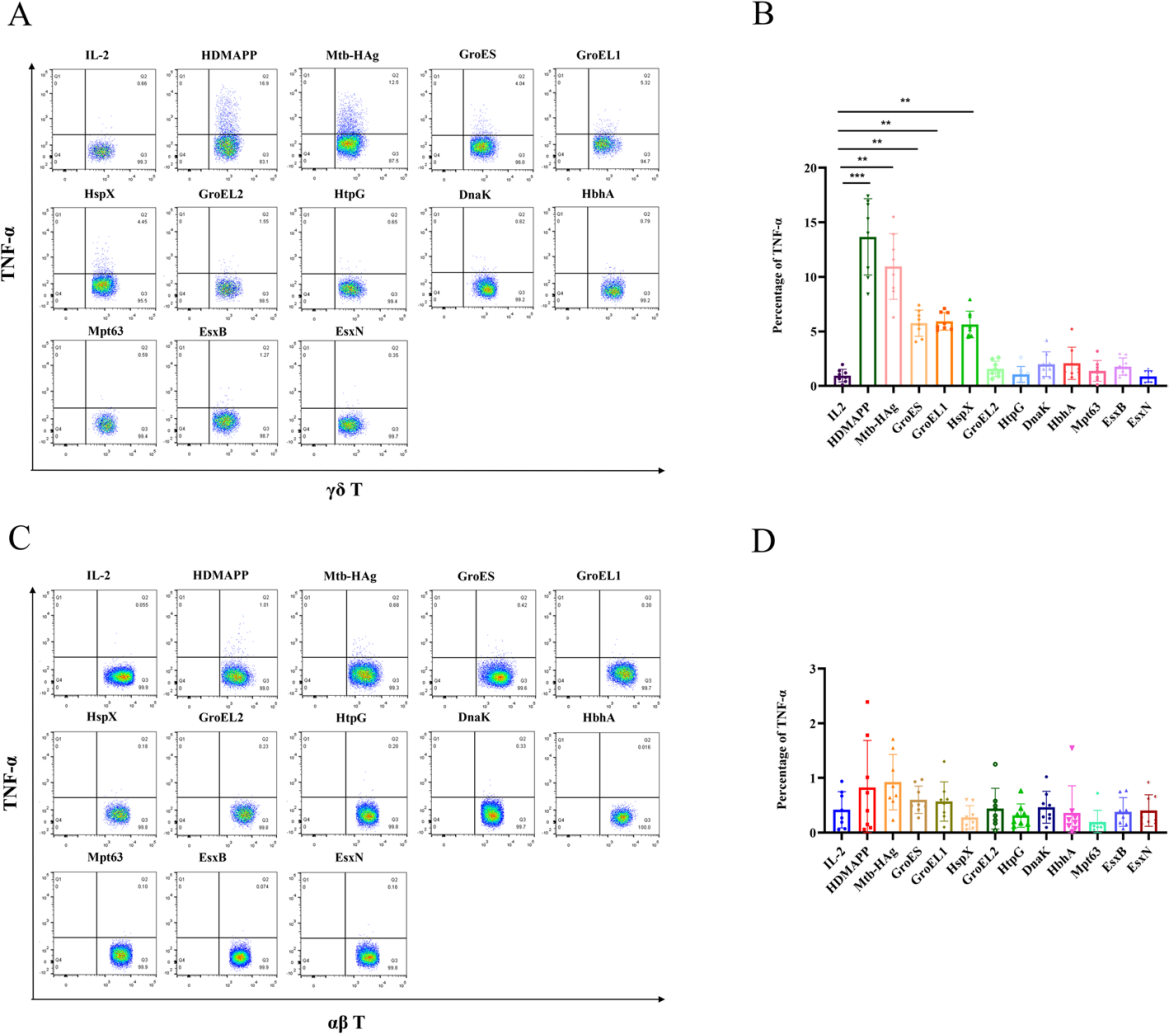
Proportion of secreted TNF-α-producing cells in Mtb-HAg and recombinant protein-stimulated peripheral blood γδ T cells and αβ T cells. **A** Flow cytometric analysis of TNF-α production by specifically expanded γδ T cells. **B** Statistical analysis of TNF-α production by specifically expanded γδ T cells (***P < 0.001, **P < 0.01). **C** Flow cytometric analysis of TNF-α production by specifically expanded αβ T cells. **D** Statistical analysis of TNF-α production by specifically expanded αβ T cells (***P < 0.001, **P < 0.01)

**Fig. 7:**
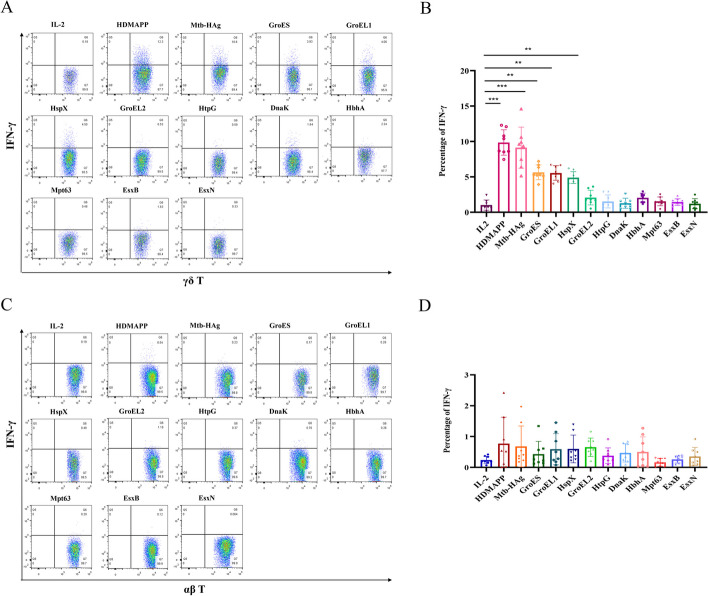
Proportion of secreted IFN-γ-producing cells in Mtb-HAg and component-stimulated peripheral blood γδ T cells and αβ T cells. **A** Flow cytometric analysis of IFN-γ production by specifically expanded γδ T cells. **B** Statistical analysis of IFN-γ production by specifically expanded γδ T cells (***P < 0.001, **P < 0.01). **C** Flow cytometric analysis of IFN-γ production by specifically expanded αβ T cells. **D** Statistical analysis of IFN-γ production by specifically expanded αβ T cells (***P < 0.001, **P < 0.01)

Next, we sought to examine whether recombinant protein-expanded γδ T cells could produce the inflammatory cytokines TNF-α and IFN-γ. We used conventional intracellular cytokine staining under antigenic stimulation in vitro. Flow cytometry was used to measure the levels of cytokine TNF-α and IFN-γproduced by γδ T cells. The results showed that the proportion of TNF-α-producing cells in GroES (5.75 ± 1.12%), GroEL1 (5.92 ± 0.76%), and HspX (5.63 ± 1.14%)-stimulated γδ T cells was significantly higher than that of the negative control IL-2 group (0.95 ± 0.55%) and was statistically significant. Cells stimulated with GroEL2 (1.56 ± 0.67%), HtpG (1.06 ± 0.68%), DnaK (1.99 ± 1.05%), HbhA (2.07 ± 1.38%), Mpt63 (1.38 ± 0.89%), EsxB (1.77 ± 0.74%), and EsxN (0.87 ± 0.49%) produced TNF-α, but the results were not statistically significant when compared to the negative control IL-2 (0.95 ± 0.55%) (Fig. 6A, B). In addition, the levels of TNF-α produced by αβ T cells stimulated with GroES (0.60 ± 0.23%), GroEL1 (0.57 ± 0.34%), HspX (0.28 ± 0.20%), GroEL2 (0.44 ± 0.35%), HtpG (0.31 ± 0.20%), DnaK (0.47 ± 0.27%), HbhA (0.36 ± 0.46%), Mpt63 (0.19 ± 0.20%), EsxB (0.38 ± 0.25%), and EsxN (0.41 ± 0.27%) were examined by flow cytometry, and the results showed that none of the 10 recombinant protein-stimulated αβ T cells produced significantly increased levels of TNF-α compared to those produced by the negative control IL-2 group (0.42 ± 0.31%) (Fig. 6C, D). As with TNF-α, the proportion of IFN-γ-producing specific γδ T cells stimulated with GroES (5.64 ± 0.98%), GroEL1 (5.55 ± 0.96%), and HspX (4.91 ± 0.78%) was significantly higher than that in the negative control IL-2 group (1.02 ± 0.67%); however, cells stimulated with GroEL2 (2.08 ± 0.97%), HtpG (1.54 ± 0.86%), DnaK (1.28 ± 0.66%), HbhA (2.07 ± 0.59%), Mpt63 (1.55 ± 0.58%), EsxB (1.45 ± 0.43%), and EsxN (1.23 ± 0.66%) could not directly induce IFN-γ production by human peripheral blood γδ T cells compared to the negative control IL-2 (1.02 ± 0.67%) (Fig. 7A, B). At the same time, the levels of the cytokine IFN-γ produced by αβ T cells were determined by flow cytometry, which showed that GroES (0.43 ± 0.39%), GroEL1 (0.59 ± 0.47%), HspX (0.60 ± 0.42%), GroEL2 (0.56 ± 0.32%), HtpG (0.38 ± 0.24%), DnaK (0.47 ± 0.27%), HbhA (0.50 ± 0.46%), Mpt63 (0.17 ± 0.12%), EsxB (0.26 ± 0.11%), and EsxN (0.36 ± 0.27%) also failed to directly induce IFN-γ production by αβ T cells compared to the negative control IL-2 (0.24 ± 0.11%), with no statistical significance (Fig. 7C, D). Thus, the ability of activated γδ T cells and the failure of αβ T cells to produce inflammatory cytokines were confirmed.

### Intracellular mycobacterial growth inhibition assay

**Fig. 8:**
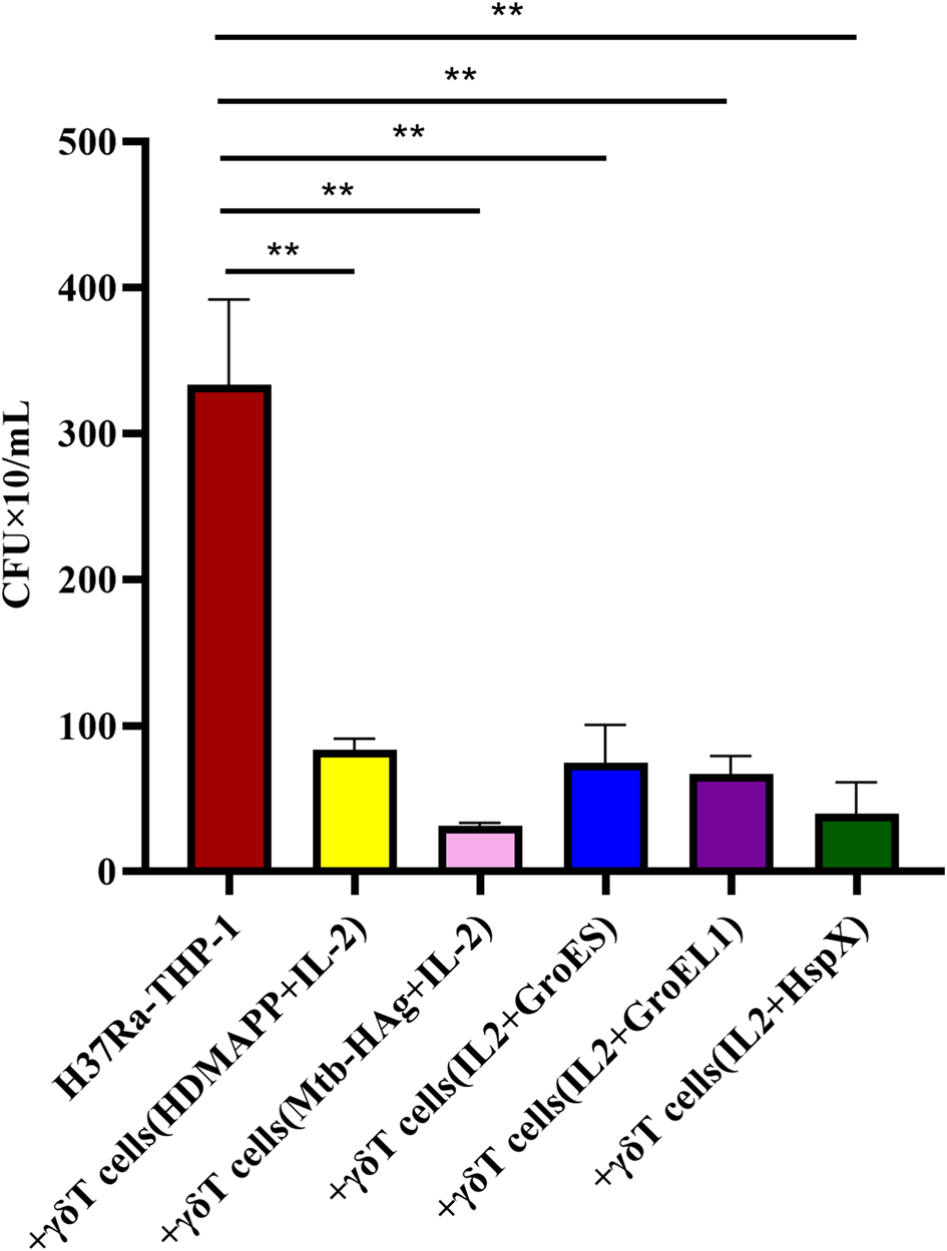
Expanded γδ T by GroES, GroEL1, HspX cells inhibit intracellular H37Ra growth in THP-1 cells, respectively (**P < 0.01)

Considering that HspX, GroEL1, GroES-expanded γδ T cells produce a variety of anti-mycobacterial cytokines, we hypothesized that expanded γδ T effector cells could limit Mtb replication during Mtb infection. To test this hypothesis, we assessed the ability of γδ T cells to regulate intracellular Mtb replication using the target of Mtb H37Ra infection. Because earlier studies have shown that activated γδ T effector cells inhibit Mtb growth more significantly than quiescent γδ T cells [8, 9], we compared the inhibitory effects of γδ T effector cells activated by GroES, GroEL1, HspX on the growth of Mtb H37Ra. Expanded γδ T cells were co-cultured with H37Ra-infected THP-1 and then evaluated for their ability to affect intracellular H37Ra growth. We found that γδ T effector cells purified from HDMAPP, Mtb-HAg, GroES, GroEL1, HspX cultures significantly inhibited the growth of Mtb H37Ra in THP-1 target cells compared to medium alone (Fig. 8). These results confirm that γδ T effector cells proliferating in HspX, GroEL1, GroES can limit intracellular Mtb growth.

## Discussion

In 2014, the WHO adopted the End TB Strategy, which aims to “reduce TB incidence by 80% and TB mortality by 90% by 2030 and eliminate the catastrophic costs to TB-affected households,” noting that a new effective vaccine is needed to achieve these goals [32]. However, despite global efforts, TB incidence has declined by only 9% cumulatively between 2015 and 2019, far short of the desired 20% reduction by 2020 [33, 34]. Therefore, there is an urgent need to develop new and more effective vaccines against TB.

Human peripheral blood T lymphocytes are divided into αβ T cells and γδ T cells based on differences in TCRs. αβ T cells are essential for the acquired immune response, whereas γδ T cells, although only a small nonconventional T-cell population, play protective roles in immunity against TB and other infections [35, 36], and act as a “bridge” between innate and adaptive immunity. It has been reported that Mtb-HAg, a low-molecular polypeptide protein extracted from mycobacterial cells, has been shown to promote the proliferation of γδ T cells [18–20]. Nevertheless, which components of Mtb-HAg specifically play a role in activating γδ T-cell proliferation? The answer has not yet been reported. Recent studies on TCRγδ ligands have focused on nonpeptide phosphorylated antigens such as isoprenyl pyrophosphate (IPP) [37], and further elucidation of the mechanism of anti-TB infection in γδ T cells requires the identification of additional Mtb-specific TCRγδ ligands. Therefore, Mtb-HAg active components were analysed and identified to clarify the mechanism of Mtb-HAg activation of γδ T-cell proliferation.

In this study, Mtb-HAg of Mtb attenuated H37Ra strain was analysed by LC‒MS mass spectrometry, from which 564 proteins were identified. Analysis of the identified protein fractions revealed that four of the top 10 scoring proteins were HSPs (GroEL2, GroEL1, HspX, and DnaK), and several functional proteins, such as multiple Mtb-specific antigenic proteins, proteins encoded by drug resistance-related genes, potential anti-TB drug target proteins, latent infection-associated proteins, and iron metabolism-associated proteins, were also identified. HSPs are a highly conserved class of proteins that are heat resistant and can resist high temperatures. HSPs of Mtb can influence TB development and progression through interactions with macrophages [38]. It was found that the expression levels of GroEL1 and GroEL2 were significantly increased under stress conditions such as oxidative stress, heat shock and infection [39, 40]. In addition, Hu et al. [41] found that GroEL2 is an essential protein for the growth of Mtb. HspX is the major antigen during the latent infection phase of Mtb and is involved in mycobacterial survival as a potent molecular chaperone that protects proteins from aggregation in vitro and serves as an antigen for the rapid diagnosis of Mtb [42–44]. The greater antigenicity of the DnaK protein can stimulate T cells to generate an immune response, which is valuable for the diagnosis of TB and the research of novel anti-TB vaccines [45], and it has been shown that overexpression of DnaK in Mtb enhances the clearance of pathogens in a mouse model [7]. The HtpG protein is a metal-dependent ATPase that plays a synergistic role with various chaperone proteins in Mtb, such as DnaK, and exerts proteostatic effects to maintain the Mtb proteome [46]. The discovery and identification of the abovementioned Mtb-HAg components lay the foundation for further studies on the mechanism by which Mtb-HAg promotes the proliferation of γδ T cells in vitro.

It is well known that γδ T cells exist only in primates and recognize phosphoantigens from specific pathogens, including Mtb, but the mechanism of Mtb-HAg recognition has been less well studied, and its role in immunity against tuberculosis and other infectious diseases has not yet been determined. Vγ2Vδ2 T cells represent the major circulating γδ T-cell subset in humans, typically accounting for 65–90% of total peripheral blood γδ T cells [47–50]. TB infection leads to a reduction in the Vδ2 subset, which may be due to the presence of specific Mtb ligands that induce sustained activation of Vδ2 T cells, followed by a subsequent reduction in this cell subset through spontaneous and activation-induced apoptosis [51, 52]. γδ T cells enhance human immunity to Mtb by secreting cytokines and promoting anti-TB immune responses elicited by other immune cells, such as macrophages [53, 54]. TNF-α is a major cytokine of the host's natural defence mechanism. TNF-α recruits innate immune cells, such as monocytes and granulocytes, to the site of infection and contributes to the formation of tuberculous granulomas in infected tissues, which helps to prevent the spread of tuberculosis bacteria in the body [55]. According to several studies, TNF-α levels tend to be high in patients with active TB infection [56]. Similarly, the cytokine IFN-γ is essential for protective defence against intracellular infection and is a key regulator of macrophage activation in Mtb infection [57, 58].

The purpose of the present study was to determine which Mtb-HAg components play a role in the induction of human peripheral blood γδ T-cell proliferation and the specific induction of antitubercular cytokine production, thus providing new insights into the role these components play in TB immunization. In vitro functional experiments showed that the components identified from Mtb-HAg, including HspX, GroEL1, and GroES, could dominantly expand γδ T cells from human peripheral blood with Vγ2Vδ2 T cells as the main cell subset and could stimulate the production of TNF-α and IFN-γ, while the proportion of TNF-α- and IFN-γ-producing cells in αβ T cells was significantly lower than that in γδ T cells, and there was no significant difference when compared with the control IL-2 group. This finding suggests that γδ T cells expanded with HspX, GroEL1, and GroES as stimulants can directly produce IFN-γ and TNF-α, similar to Mtb-HAg [22], and at the same time, since these three recombinantly expressed proteins do not directly activate αβ T cells, they cannot specifically induce the production of TNF-α or IFN-γ in these cells.

Some studies have shown that resting γδ T cells expanded in vitro by live Mtb were specific to Mtb and that heat killing and washing the mycobacteria removed the antigen(s) for γδ T cells [52, 59, 60]. In contrast, heat-killed mycobacteria retained significant antigenicity for CD4^+^ T cells [61]. The antigen repertoire and HLA requirements for CD4^+^ memory T cells and those for γδ T cells appear to be quite distinct. CD4^+^ T cells recognize both soluble protein antigens and whole organisms in a class II major histocompatibility complex-restricted manner, whereas γδ T cells appear to recognize only constituents associated with the whole organism and are not restricted by class I or class II major histocompatibility complex molecules [62]. γδ T cells might be part of the primary response to infection with live bacteria, whereas CD4^+^ T cells regulate the secondary immune response, which protects against exogenous reinfection or reactivation of endogenous disease. αβ T cells are not sensitive to Mtb-HAg; the total number of αβT cells was only slightly increased, but the percentage of αβ T cells was relatively decreased [24].

Early human studies have shown that phosphoantigen and IL-12 effectively stimulate human Vγ2Vδ2 T cells in vitro, causing these highly activated γδ T cells to lyse *mycobacterium*-infected cells or inhibit the growth of Mtb [30, 31]. However, Spencer et al. [63] found that phosphoantigen-expanded γδ T cells could develop similar effector and cytolytic functional capacities, but failed to inhibit the growth of mycobacterium. Thus, the relationship between the proportion of phosphoantigen-expanded γδ T cells and their ability to resist *mycobacterium* growth remains controversial. The present study further explores the active components of Mtb-HAg and demonstrates that they can stimulate the proliferation of γδ T effector cells and the production of TNF-α and IFN-γ by these cells. The ability of γδ T cells induced by different recombinant proteins to simultaneously produce IFN-γ and TNF-α is consistent with earlier reports that γδ T cells have a pleiotropic ability to produce multiple cytokines [31, 36, 64]. The correlation between anti-TB immunity and coproduction of IFN-γ and TNF-α by γδ T cells is consistent with previous observations that both IFN-γ and TNF-α are involved in the ability of γδ T effector cells to inhibit intracellular Mtb spread [30]. Our findings support the possibility that γδ T effector cells and anti-TB cytokines may contribute to Mtb-HAg-induced resistance to Mtb infection or TB lesions.

Therefore, the present study provides new information demonstrating that immunostimulation with the Mtb-HAg proteins HspX, GroEL1, and GroES significantly promotes the expansion of γδ T cells and the production of anti-tuberculosis cytokines, such as TNF-α and IFN-γ, in the early stages of Mtb infection. These properties provide a theoretical basis for the design of subsequent TB therapeutic studies and strong support for further preclinical and clinical development of TB preventive vaccines. Notably, vaccination approaches that enhance γδ T-cell responses may provide better protection against Mtb attacks than the standard Mycobacterium bovis Bacillus Calmette-Guérin (BCG) *vaccine.* The findings of this study strongly suggest that Mtb-HAg-induced γδ T cells should be considered relevant targets for the design and development of new TB vaccines and immunotherapies. However, there are some limitations in this study. For example, the protein screened using LC‒MS was obtained by recombinant expression through genetic engineering techniques and does not have the protective immunity conferred by the natural form [65]. The study was limited to in vitro healthy human specimens only, the number of observed cases was small, and no in vivo TB model or tuberculosis patient specimens were investigated in depth.

## Conclusions

HspX, GroEL1, and GroES, active components of Mtb-HAg, specifically induced the expansion of a large number of γδ T cells, of which Vγ2δ2 T cells were the major subset, and promoted the secretion of the anti-TB cytokines TNF-α and IFN-γ. In contrast, HtpG, DnaK, GroEL2, HbhA, Mpt63, EsxB, and EsxN could not promote γδ T-cell proliferation and the secretion of TNF-α and IFN-γ, and none of the 10 different recombinant proteins mentioned above could induce αβ T cells to secrete the intracellular cytokines TNF-α and IFN-γ. Activated γδ T cells induced by Mtb-HAg components HspX, GroES, GroEL1 to produce TNF-α, IFN-γ modulate macrophages to inhibit intracellular Mtb growth.

### Supplementary Information

Additional file 1: Table S1. Primers sequences used to obtain 12 target genes by PCR amplification.

Additional file 2: Table S2. 564 protein fractions were obtained after 0.05 filtration-identification and quantification by LC–MS mass spectrometry.

Additional file 3: Figure S1. Identification of recombinant plasmids by colony PCR. M: DNA marker; 1–3: colony PCR products. (A) HtpG; (B) DnaK; (C) GroEL2; (D) GroEL1; (E) HspX; (F) GroES; (G) HbhA; (H) Mpt63; (I) EsxB; (J) EsxJ; (K) EsxA; (L) EsxN.

Additional file 4: DNA sequencing.

Additional file 5: Figure S2. The recombinant protein was purified by passing through Ni–NTA agarose resin. M: protein marker; 1: before purification; 2: flow-through solution; 3–5: elution solution. (A) HtpG; (B) DnaK; (C) GroEL2; (D) GroEL1; (E) HspX; (F) GroES; (G) HbhA; (H) Mpt63; (I) EsxB; (J) EsxN.

Additional file 6: Figure S3. Flow cytometric analysis of Vγ2Vδ2 T cells.

## Data Availability

All data generated or analyzed during this study are included in this article (and its additional files).

## Abbreviations

Mtb-HAg: *Mycobacterium tuberculosis* Heat-resistant antigen
TB: Tuberculosis
Mtb: *Mycobacterium tuberculosis*
MDR-TB: Multidrug-resistant tuberculosis
XDR-TB: Extensively drug-resistant tuberculosis
HIV: Human immunodeficiency virus
LTBI: Latent tuberculosis infection
PBMCs: Peripheral blood mononuclear cells
MHC: Major histocompatibility complex
APC: Antigen presenting cell
LC–MS: Liquid chromatography coupled to mass spectrometry
HSPs: Heat shock proteins
HMBPP: (E)-4-hydroxy-3-methyl-but-2-enyl pyrophosphate
Ni-NTA: Nitrilotriacetic acid
TCR: T cell receptors
IPP: Isoprenyl pyrophosphate
