# Supplementary material for: Analysis of the components of Mycobacterium tuberculosis heat-resistant antigen (Mtb-HAg) and its regulation of γδ T-cell function
Source: Cell Mol Biol Lett. 2024 May 13;29:70. doi: 10.1186/s11658-024-00585-7 (PMC11089708; doi:10.1186/s11658-024-00585-7)
Supplement: Supplementary file 1 — Additional file 1: Table S1. Primers sequences used to obtain 12 target genes by PCR amplification. [file 11658_2024_585_MOESM1_ESM.docx]

| **Table S1 Primers sequences used to obtain 12 target gene by PCR amplification** | | |
| --- | --- | --- |
| **Gene Symbol** |  | **Primer Sequences** |
| **pET28a-HtpG** | Forward primer(5->3) | AAGAAGGAGATATACCATGGGC ATGAACGCCCATGTCGAGCAGT |
|  | Reverse primer(5->3) | TGGTGGTGGTGGTGCTCGAG CAAGGTACGCGCGAGACGTTC |
| **pET28a-DnaK** | Forward primer(5->3) | AAGAAGGAGATATACCATGGGC ATGGCTCGTGCGGTCGGGATCG |
|  | Reverse primer(5->3) | TGGTGGTGGTGGTGCTCGAG CTTGGCCTCCCGGCCGTCGTC |
| **pET28a-GroEL2** | Forward primer(5->3) | AAGAAGGAGATATACCATGGGC ATGGCCAAGACAATTGCGTACG |
|  | Reverse primer(5->3) | TGGTGGTGGTGGTGCTCGAG GAAATCCATGCCACCCATGTC |
| **pET28a- GroEL1** | Forward primer(5->3) | AAGAAGGAGATATACCATGGGC ATGAGCAAGCTGATCGAATACG |
|  | Reverse primer(5->3) | TGGTGGTGGTGGTGCTCGAG GTGCGCGTGCCCGTGGTGATG |
| **pET28a-HspX** | Forward primer(5->3) | AAGAAGGAGATATACCATGGGC ATGGCCACCACCCTTCCCGTTC |
|  | Reverse primer(5->3) | TGGTGGTGGTGGTGCTCGAG GTTGGTGGACCGGATCTGAAT |
| **pET28a-GroES** | Forward primer(5->3) | AAGAAGGAGATATACCATGGGC ATGGTGGCGAAGGTGAACATCA |
|  | Reverse primer(5->3) | TGGTGGTGGTGGTGCTCGAG CTTGGAAACGACGGCCAGCAC |
| **pET28a- HbhA** | Forward primer(5->3) | AAGAAGGAGATATACCATGGGC ATGGCTGAAAACTCGAACATTG |
|  | Reverse primer(5->3) | TGGTGGTGGTGGTGCTCGAG CTTCTGGGTGACCTTCTTGGC |
| **pET28a- Mpt63** | Forward primer(5->3) | AAGAAGGAGATATACCATGGGC ATGAAGCTCACCACAATGATCA |
|  | Reverse primer(5->3) | TGGTGGTGGTGGTGCTCGAG CGGCTCCCAAATCAGCAGATC |
| **pET28a- EsxB** | Forward primer(5->3) | AAGAAGGAGATATACCATGGGC ATGGCAGAGATGAAGACCGATG |
|  | Reverse primer(5->3) | TGGTGGTGGTGGTGCTCGAG GAAGCCCATTTGCGAGGACAG |
| **pET28a- EsxJ** | Forward primer(5->3) | AAGAAGGAGATATACCATGGGC ATGGCCTCGCGTTTTATGACGG |
|  | Reverse primer(5->3) | TGGTGGTGGTGGTGCTCGAG GCTGCTGAGGATCTGCTGGGA |
| **pET28a- ExsA** | Forward primer(5->3) | AAGAAGGAGATATACCATGGGCATGACAGAGCAGCAGTGGAATT |
|  | Reverse primer(5->3) | TGGTGGTGGTGGTGCTCGAG TGCGAACATCCCAGTGACGTT |
| **pET28a- EsxN** | Forward primer(5->3) | AAGAAGGAGATATACCATGGGC ATGACGATTAATTACCAGTTCG |
|  | Reverse primer(5->3) | TGGTGGTGGTGGTGCTCGAG GGCCCAGCTGGAGCCGACGGC |
