# Supplementary figures and images for: Analysis of the components of Mycobacterium tuberculosis heat-resistant antigen (Mtb-HAg) and its regulation of γδ T-cell function
Source: Cell Mol Biol Lett. 2024 May 13;29:70. doi: 10.1186/s11658-024-00585-7 (PMC11089708; doi:10.1186/s11658-024-00585-7)

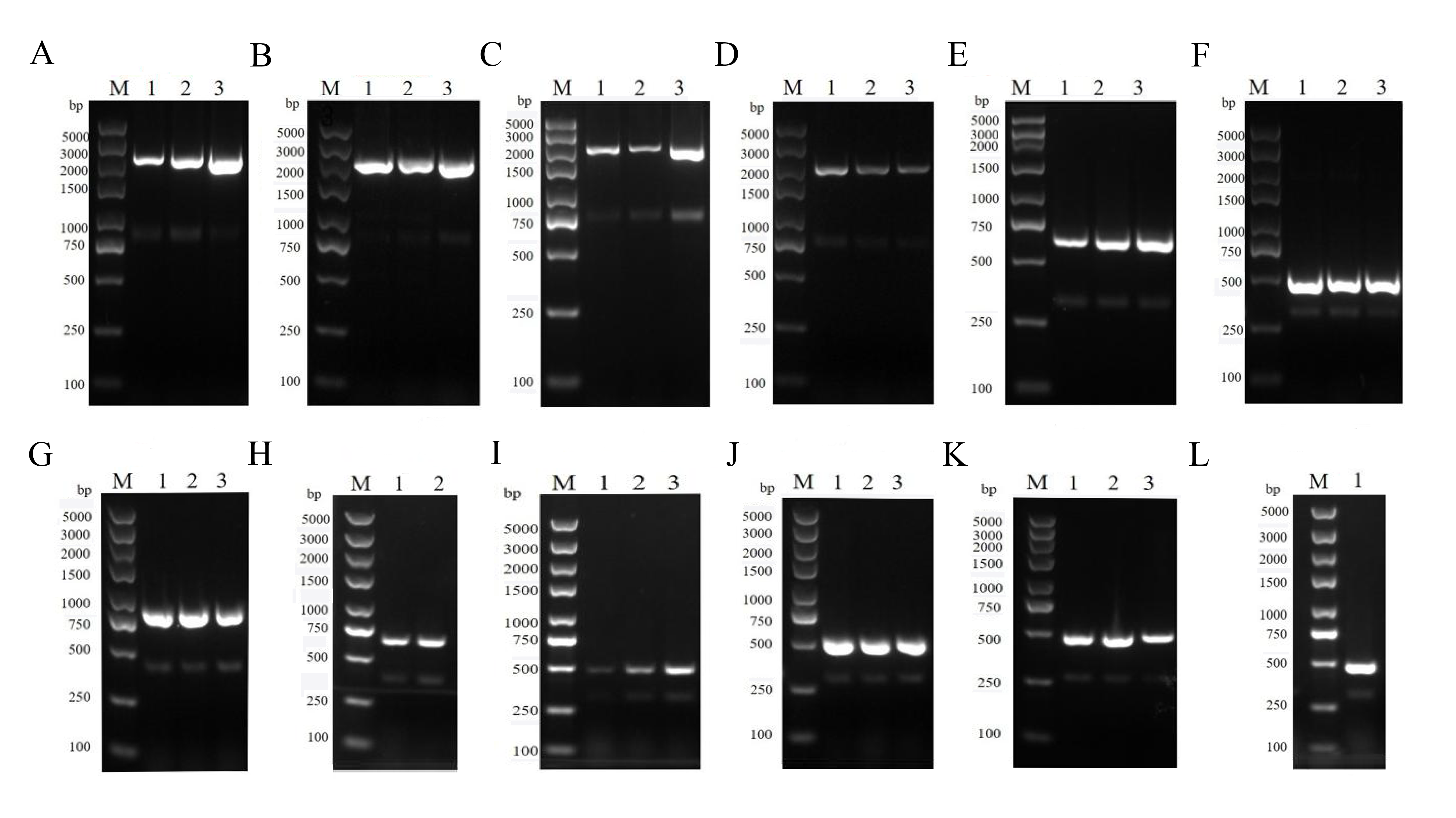

Supplement: Supplementary file 3 — Additional file 3: Figure S1. Identification of recombinant plasmids by colony PCR. M: DNA marker; 1–3: colony PCR products. (A) HtpG; (B) DnaK; (C) GroEL2; (D) GroEL1; (E) HspX; (F) GroES; (G) HbhA; (H) Mpt63; (I) EsxB; (J) EsxJ; (K) EsxA; (L) EsxN. [file 11658_2024_585_MOESM3_ESM.tif]

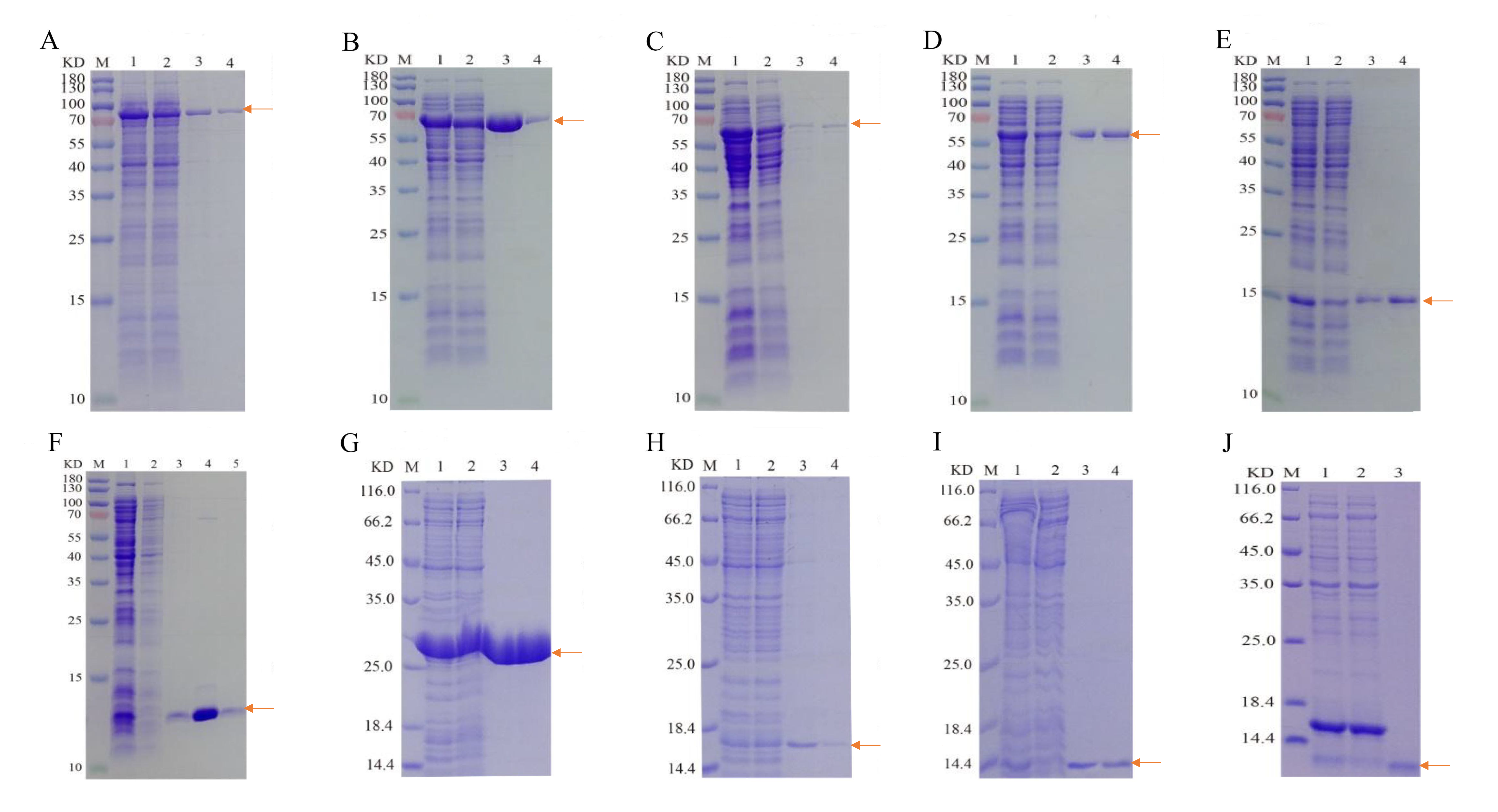

Supplement: Supplementary file 5 — Additional file 5: Figure S2. The recombinant protein was purified by passing through Ni–NTA agarose resin. M: protein marker; 1: before purification; 2: flow-through solution; 3–5: elution solution. (A) HtpG; (B) DnaK; (C) GroEL2; (D) GroEL1; (E) HspX; (F) GroES; (G) HbhA; (H) Mpt63; (I) EsxB; (J) EsxN. [file 11658_2024_585_MOESM5_ESM.tif]

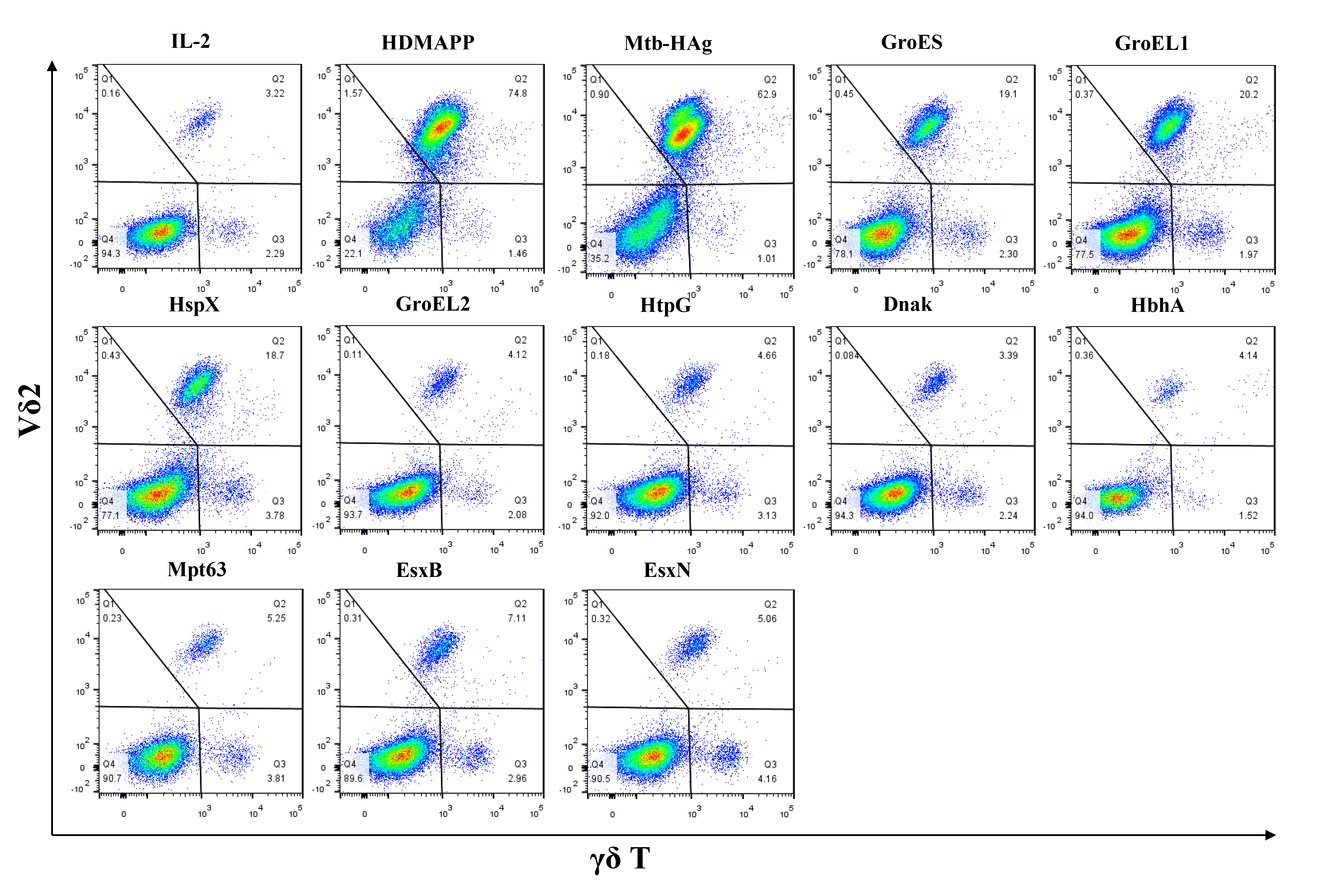

Supplement: Supplementary file 6 — Additional file 6: Figure S3. Flow cytometric analysis of Vγ2Vδ2 T cells. [file 11658_2024_585_MOESM6_ESM.tif]
