# Supplementary material for: Analysis of the components of Mycobacterium tuberculosis heat-resistant antigen (Mtb-HAg) and its regulation of γδ T-cell function
Source: Cell Mol Biol Lett. 2024 May 13;29:70. doi: 10.1186/s11658-024-00585-7 (PMC11089708; doi:10.1186/s11658-024-00585-7)

Additional file 4 : DNA sequencing

GroEL1 (MRA_3457) gene length: 1617 bp

GroEL1 (1-600 bp)

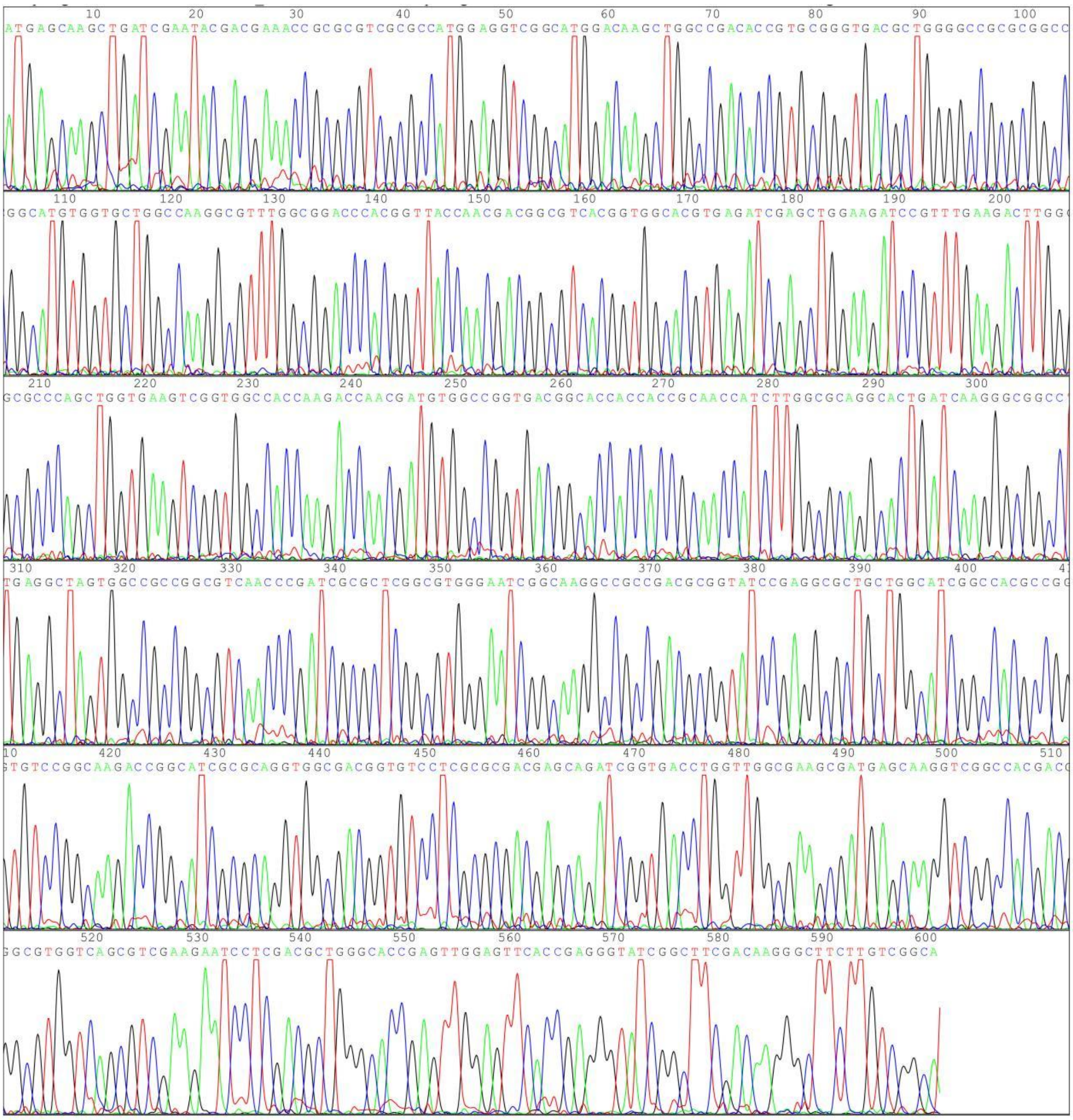

GroEL1 (601-1000 bp)

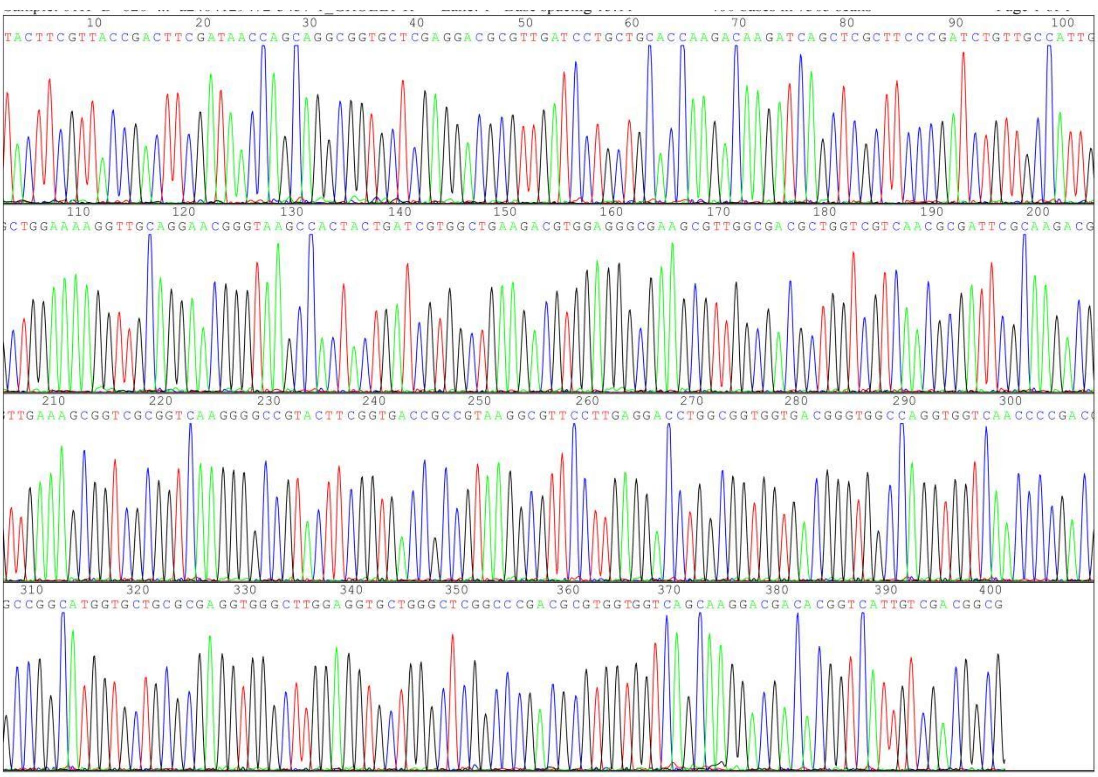

GroEL1 (1001-1617 bp)

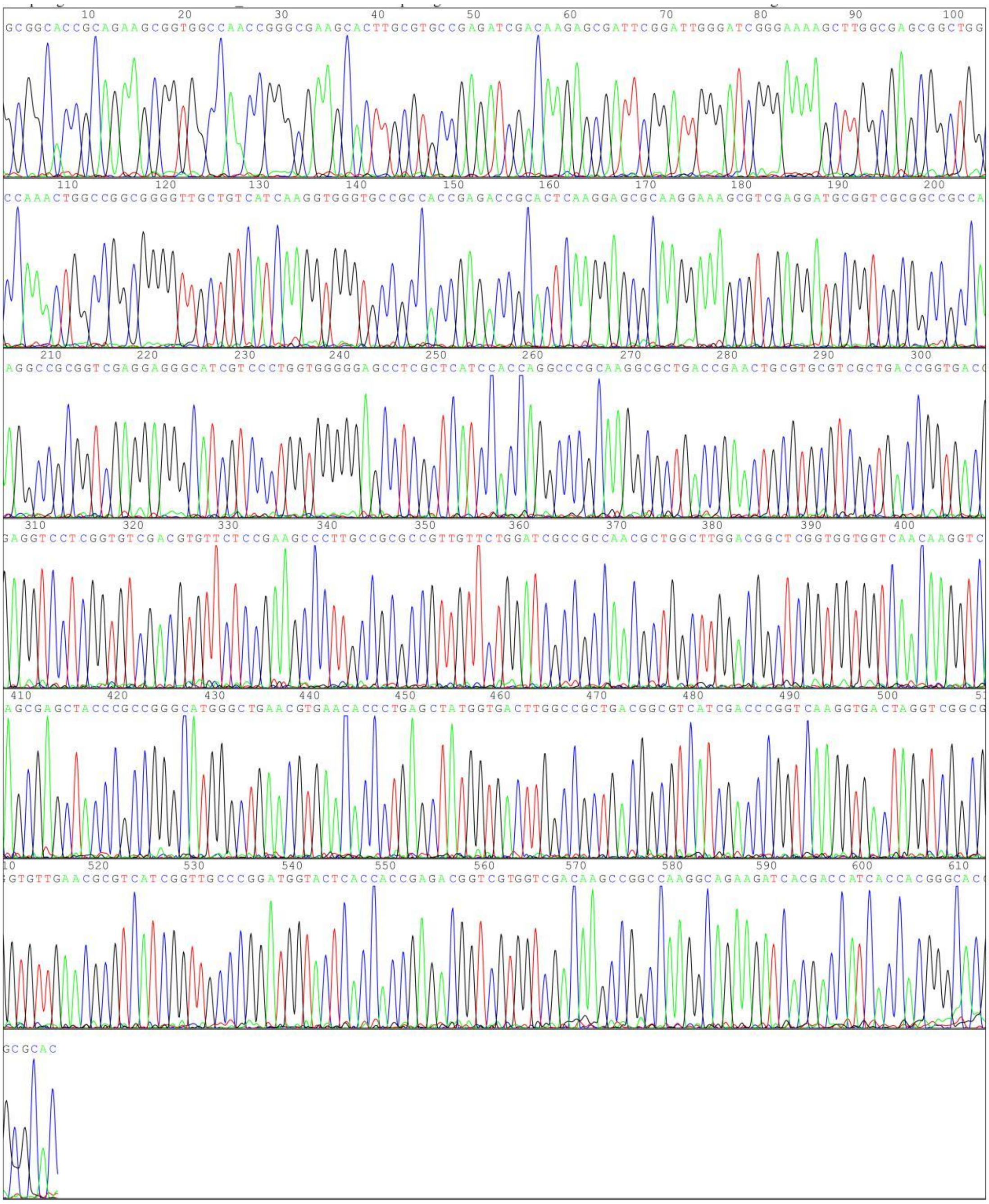

GroEL2 (MRA_0445) gene length: 1620 bp

GroEL2 (1-600 bp)

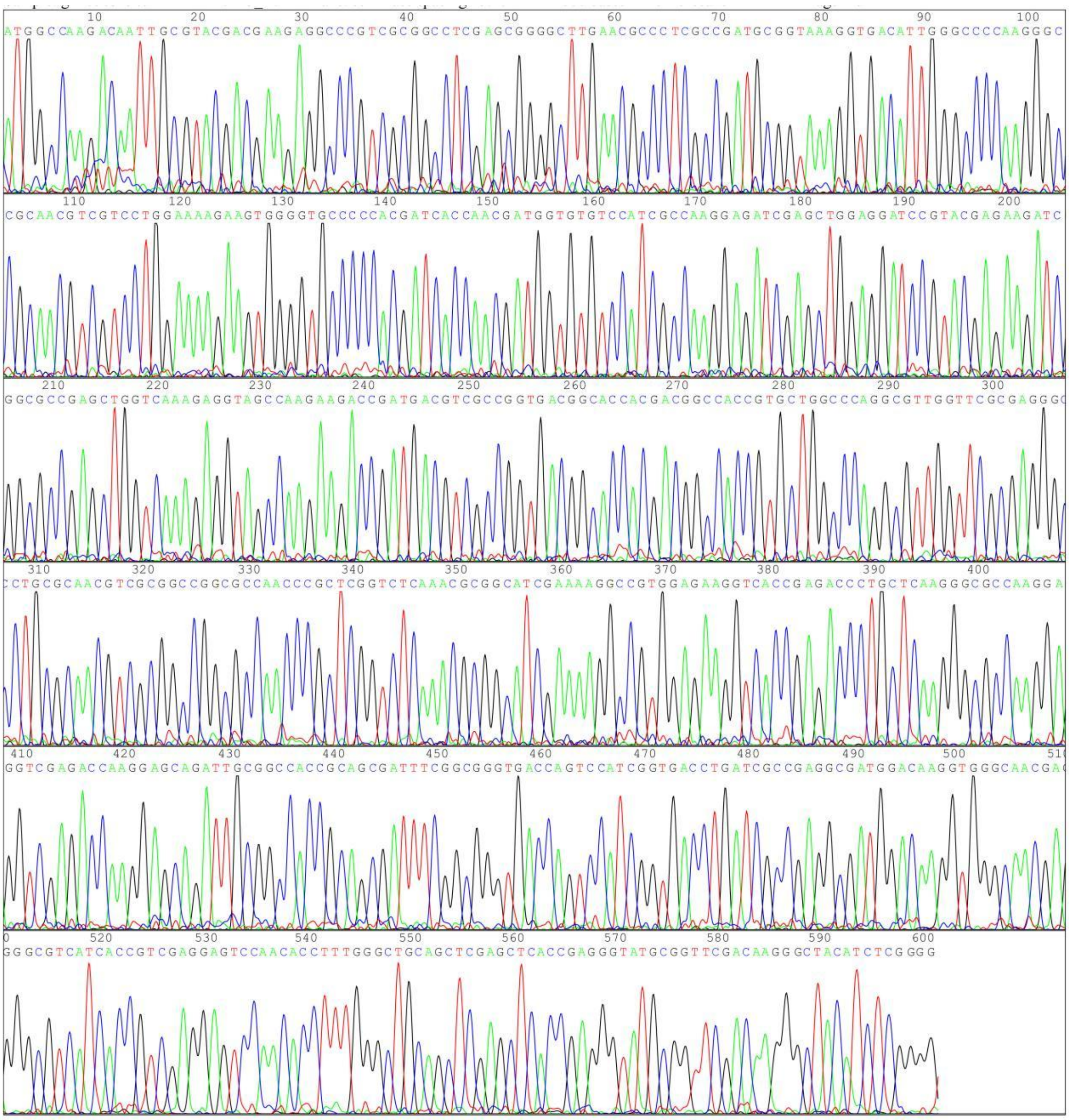

GroEL2 (601-1000 bp)

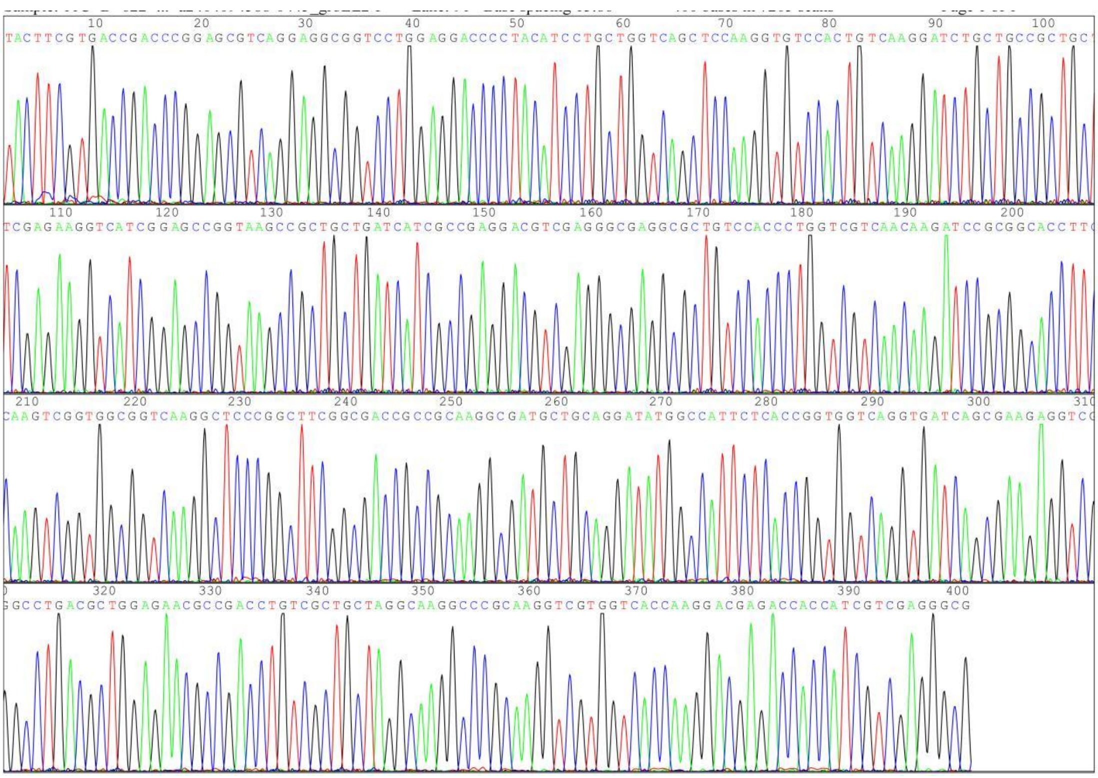

GroEL2 (1001-1620 bp)

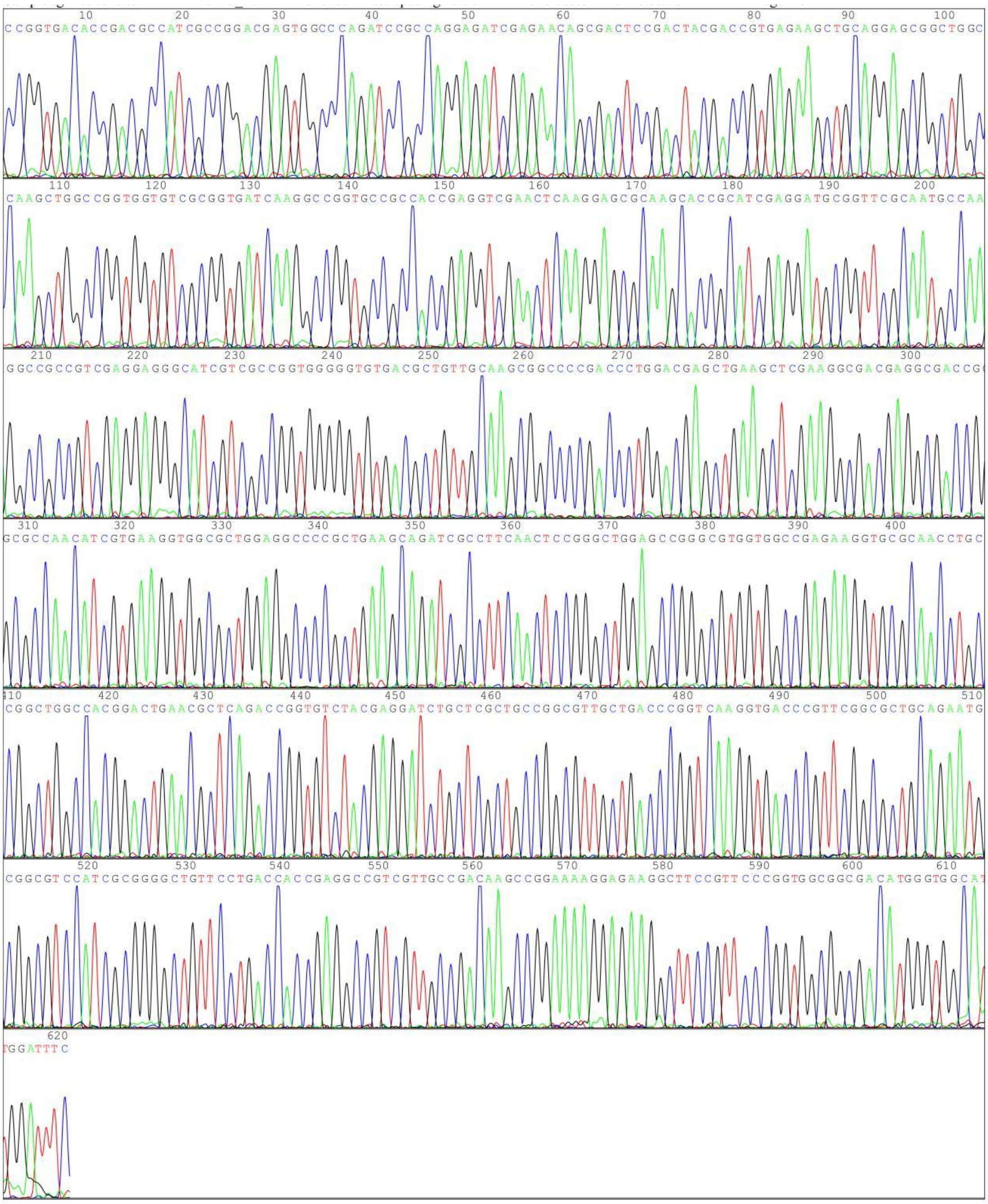

DnaK (MRA_0359) gene length: 1875bp

DnaK (1-600 bp)

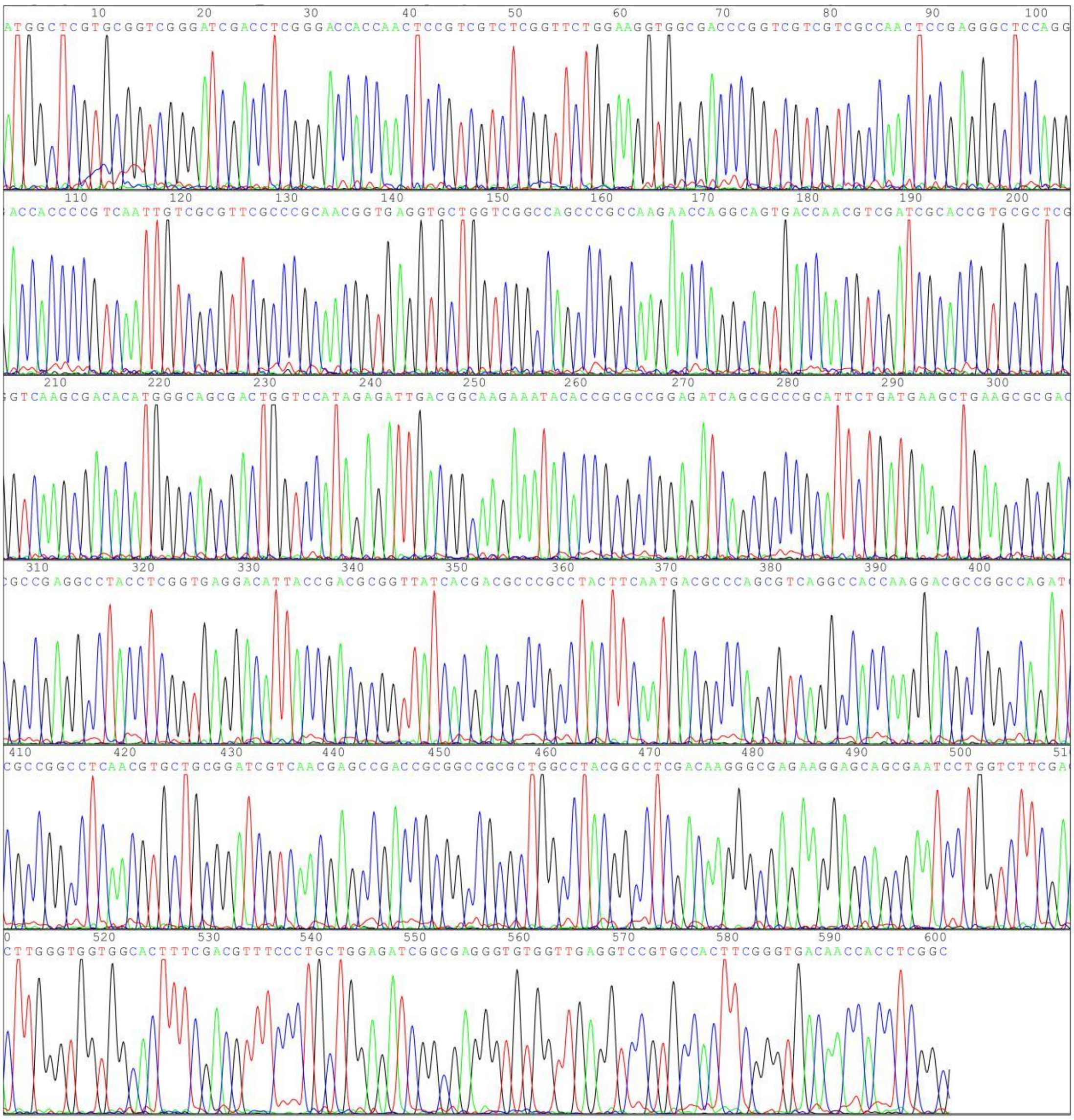

DnaK (601-1150 bp)

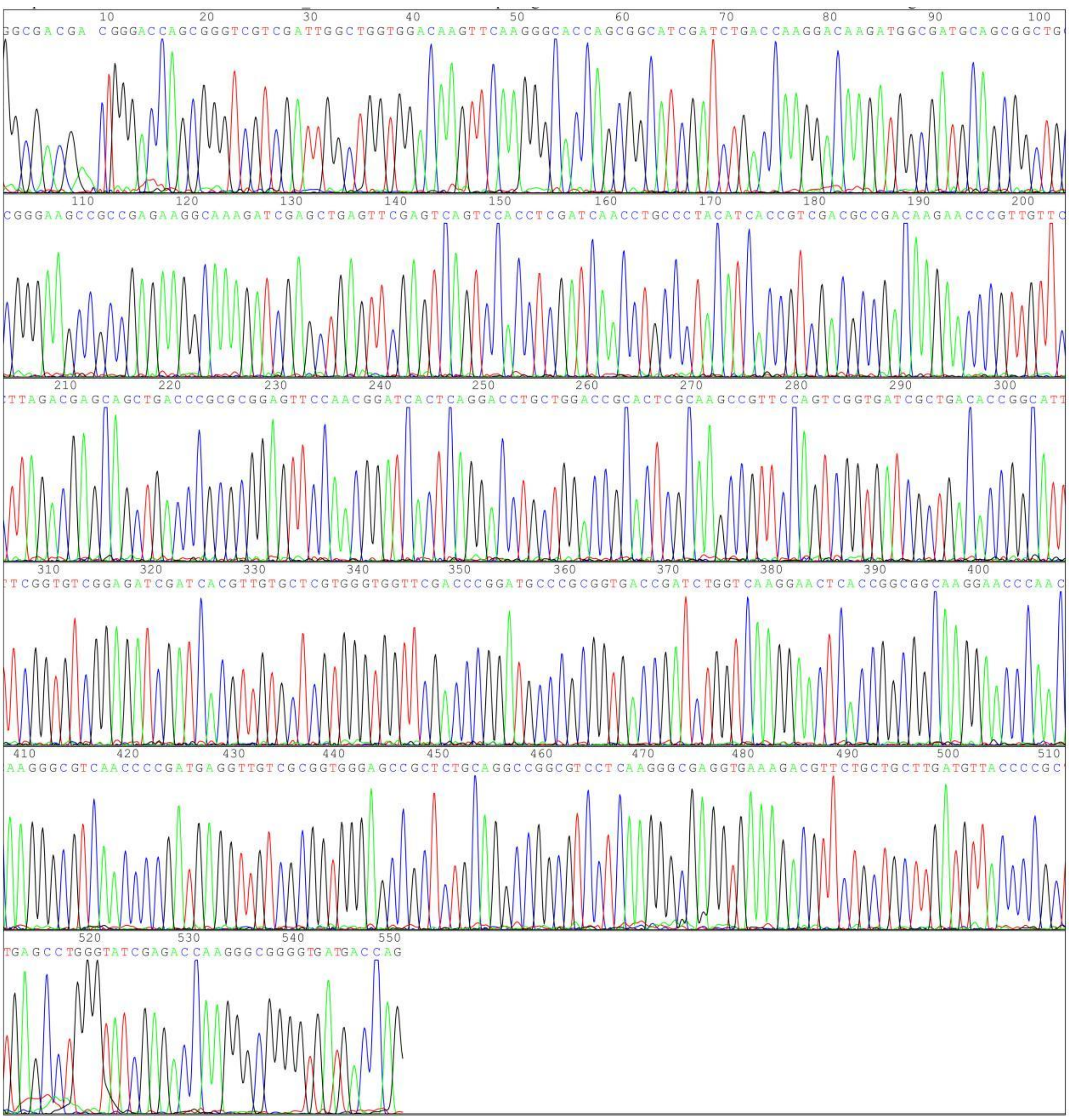

DnaK (1151-1875 bp)

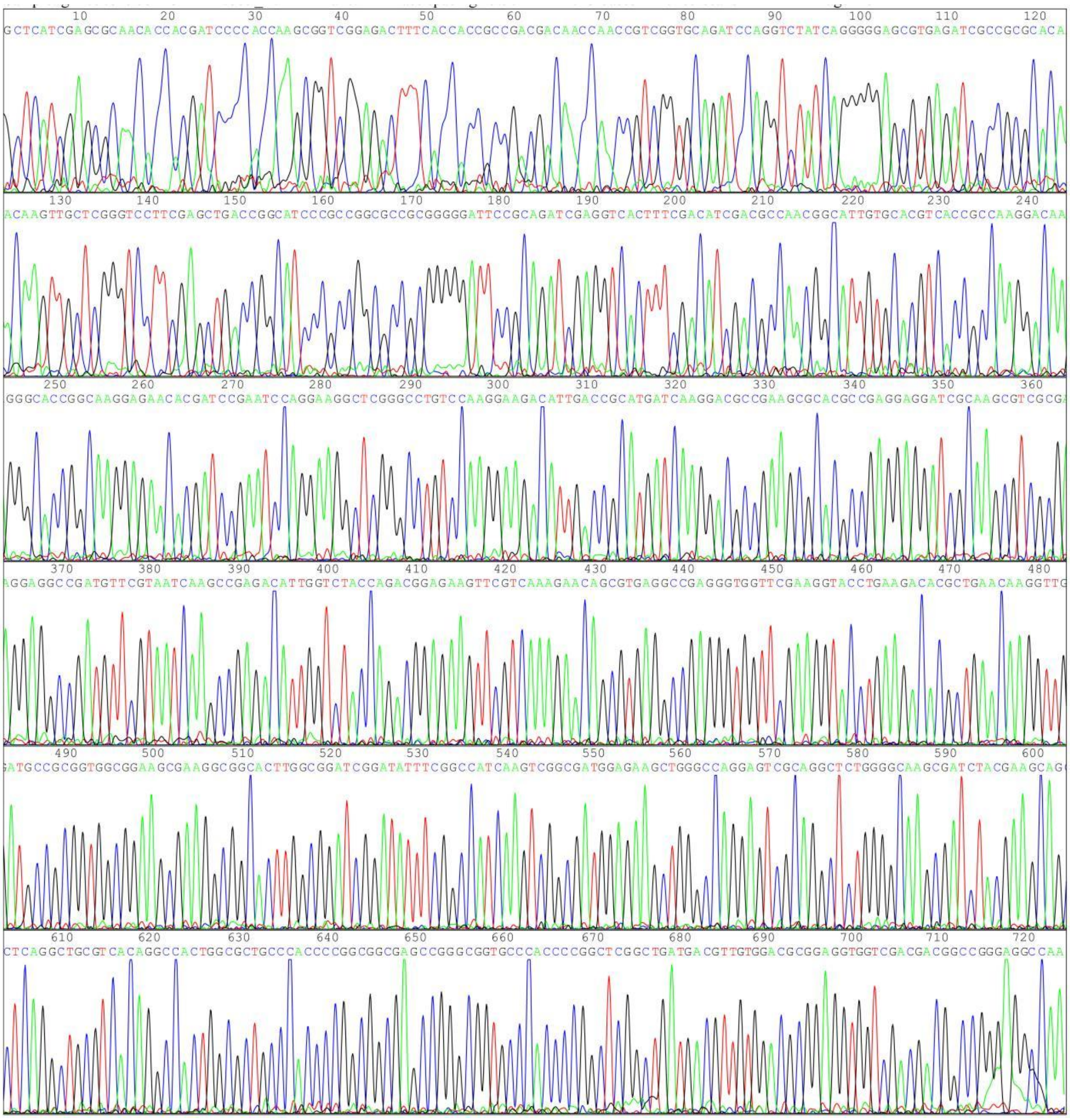

HtpG (MRA_2316) gene length: 1941bp

HtpG (1-700 bp)

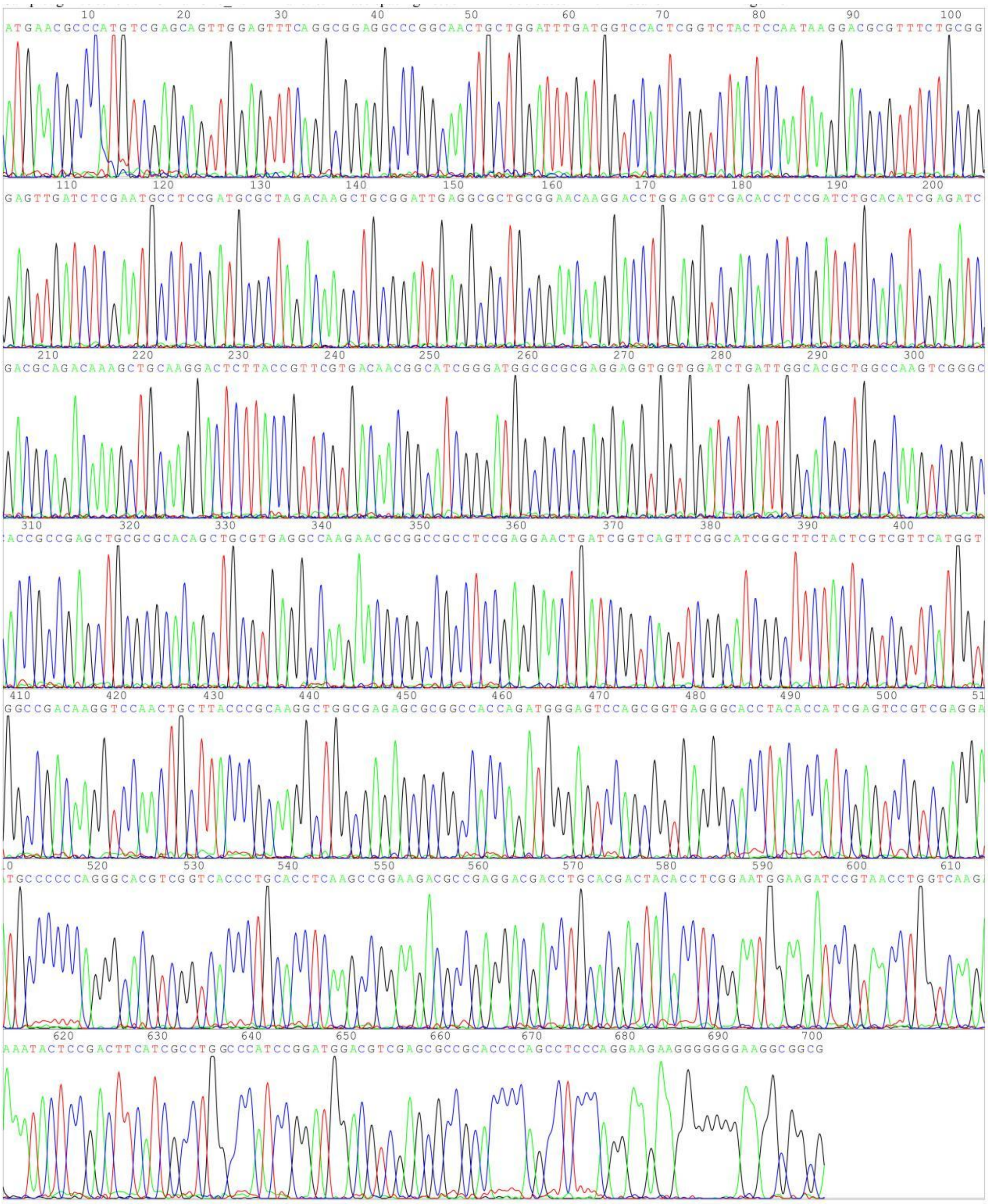

HtpG (701-1300 bp)

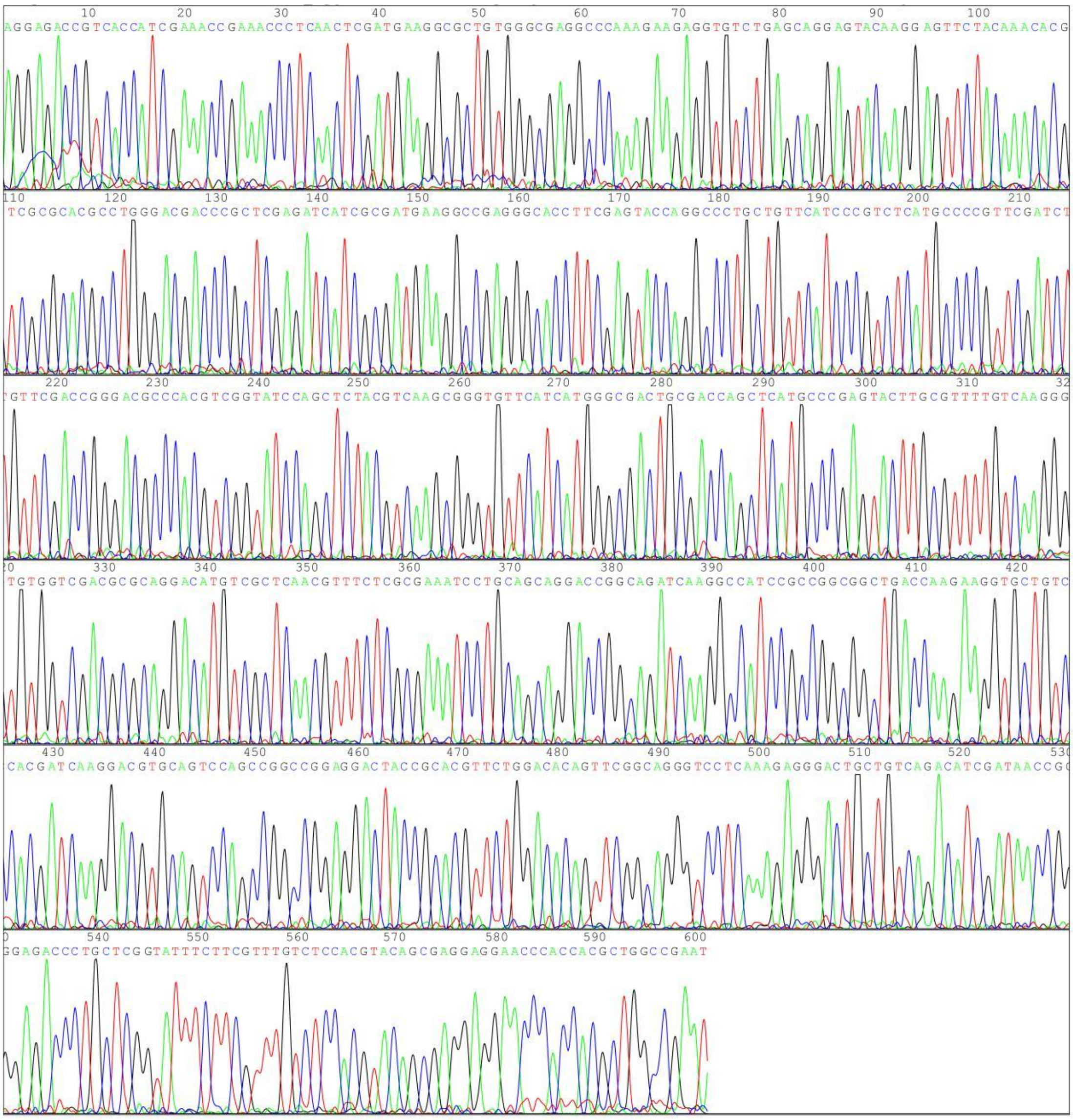

HtpG (1300-1941 bp)

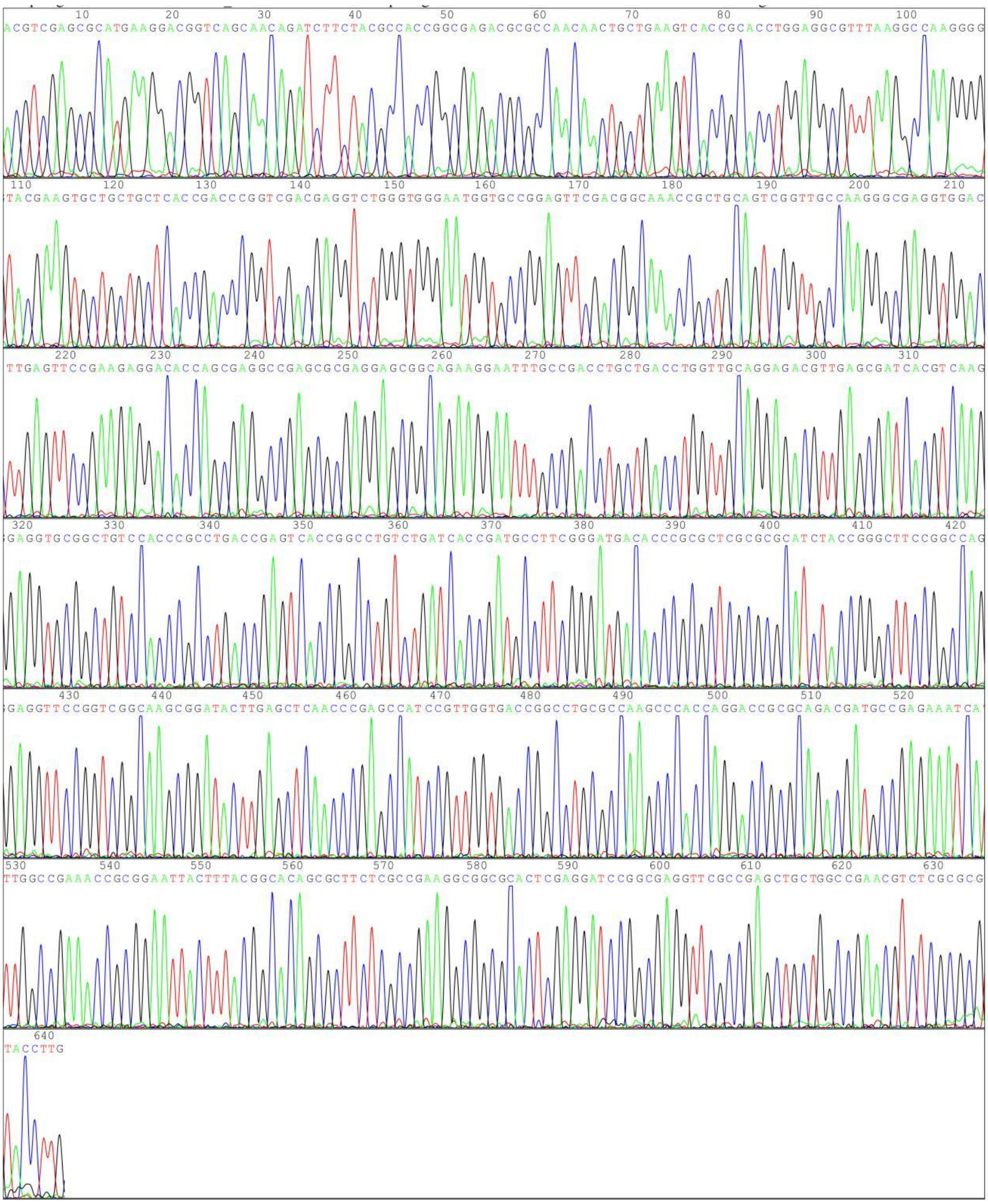

GroES (MRA_3458) gene length: 303 bp

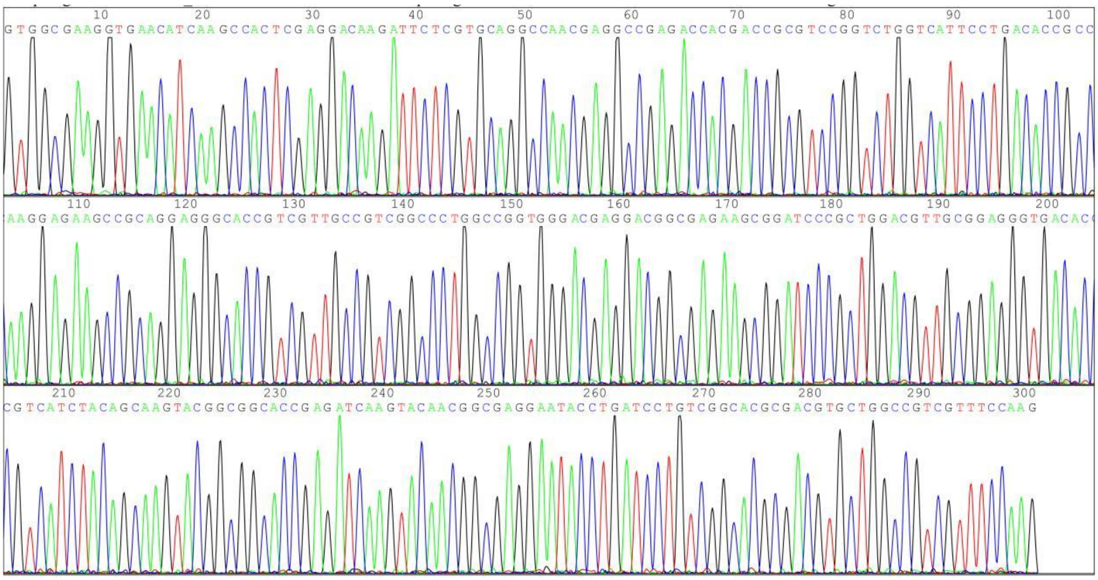

HspX(MRA_2046) gene length: 435 bp

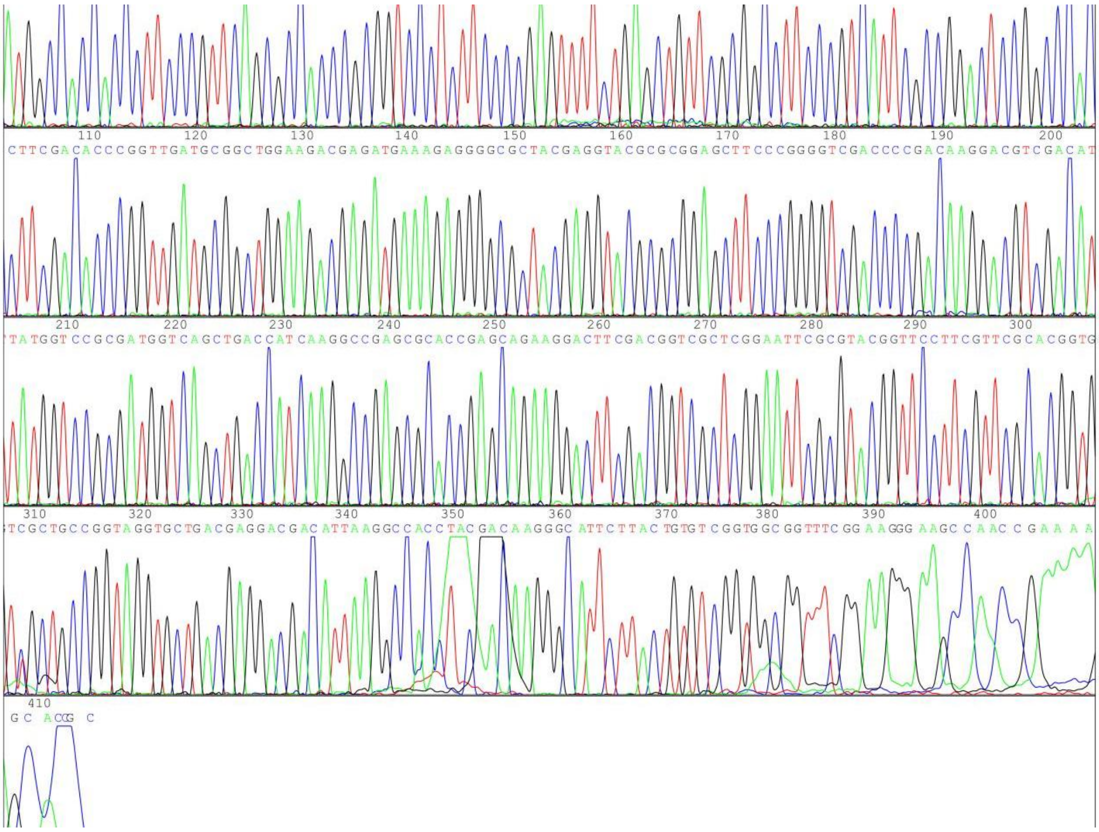

HbhA (MRA_0482) gene length: 600 bp

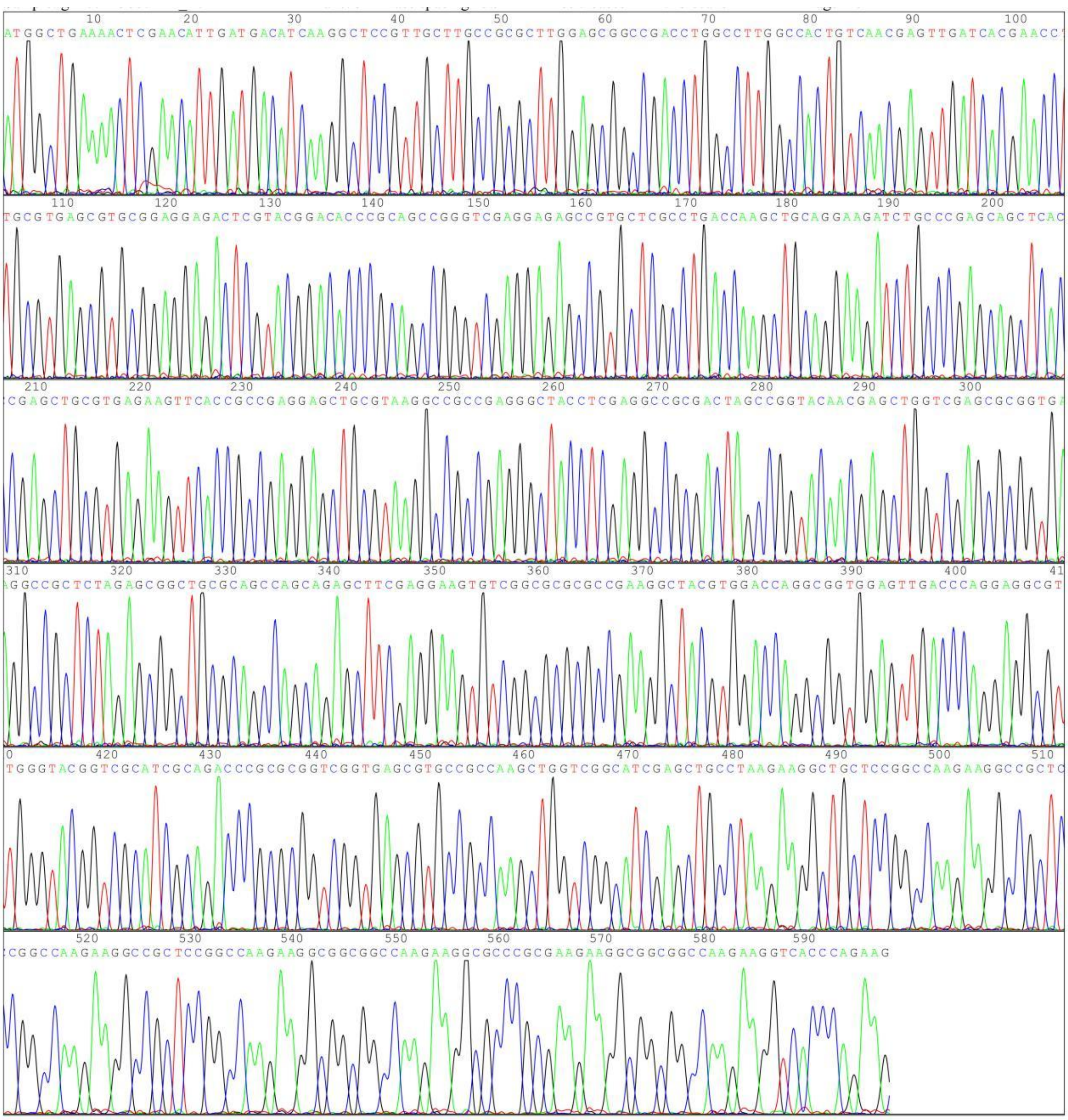

Mpt63 (MRA_1937) gene length: 480 bp

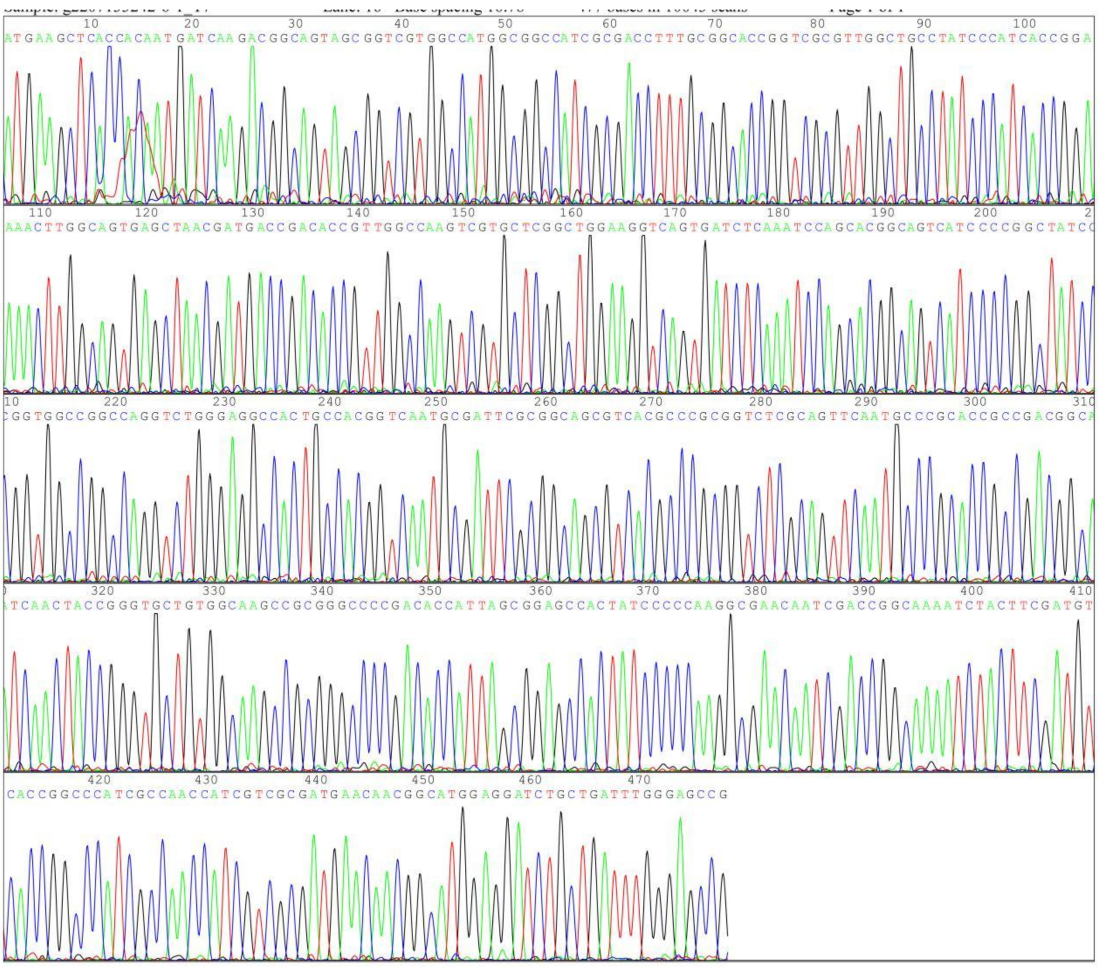

EsxB (MRA_3913) gene length: 303 bp

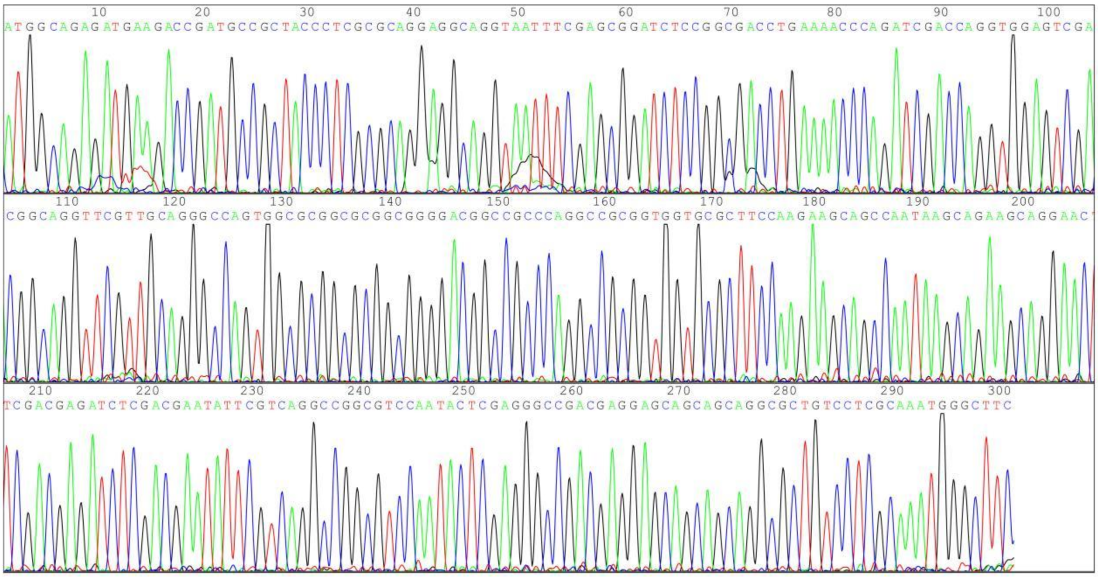

EsxJ (MRA_1046) gene length: 297 bp

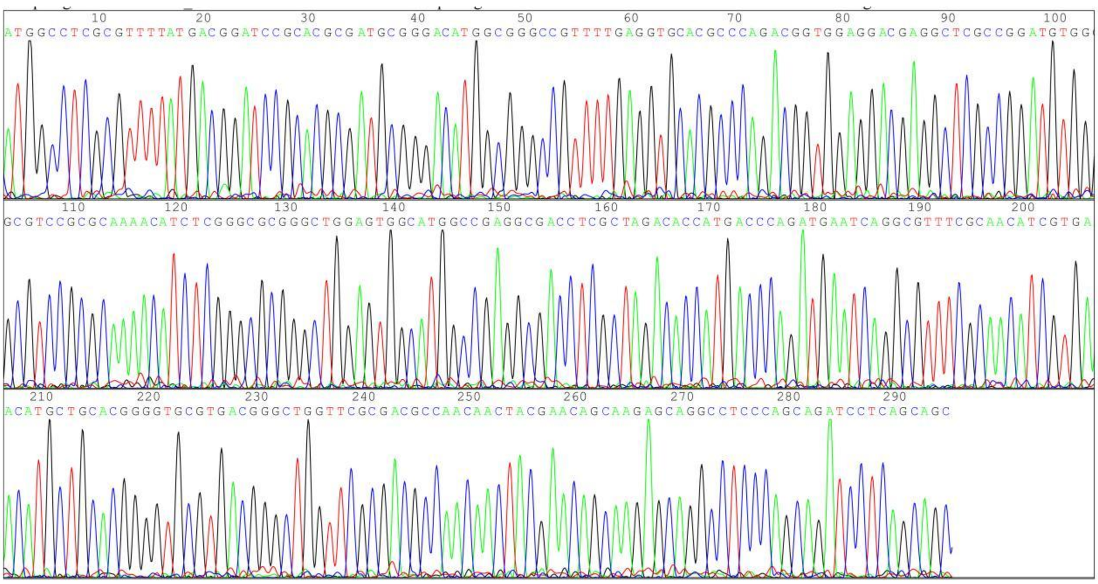

EsxA (MRA_3914)   gene length: 288 bp

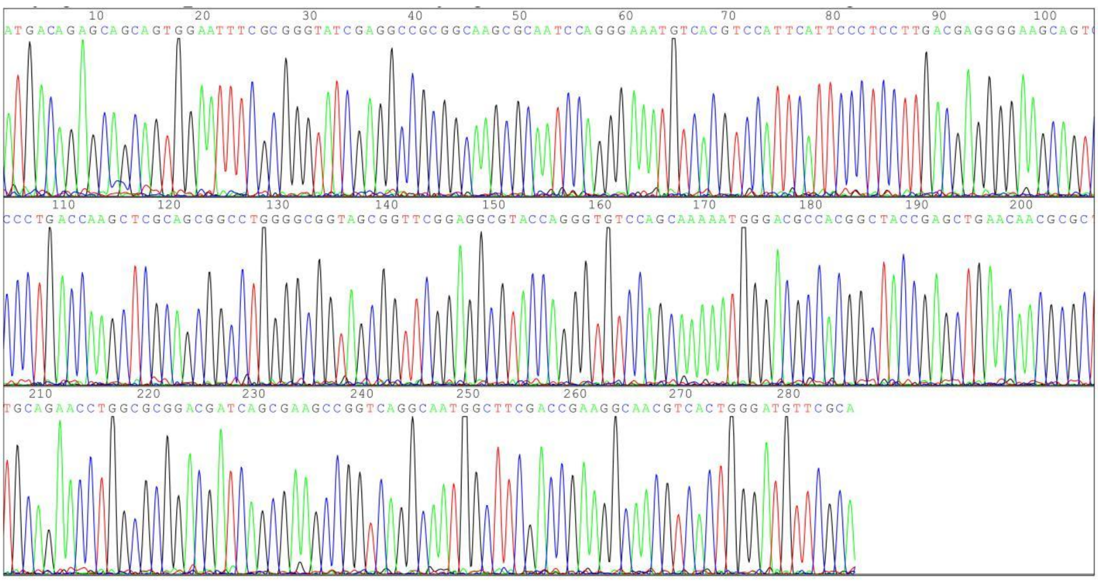

EsxN(MRA_1806)   gene length: 285 bp

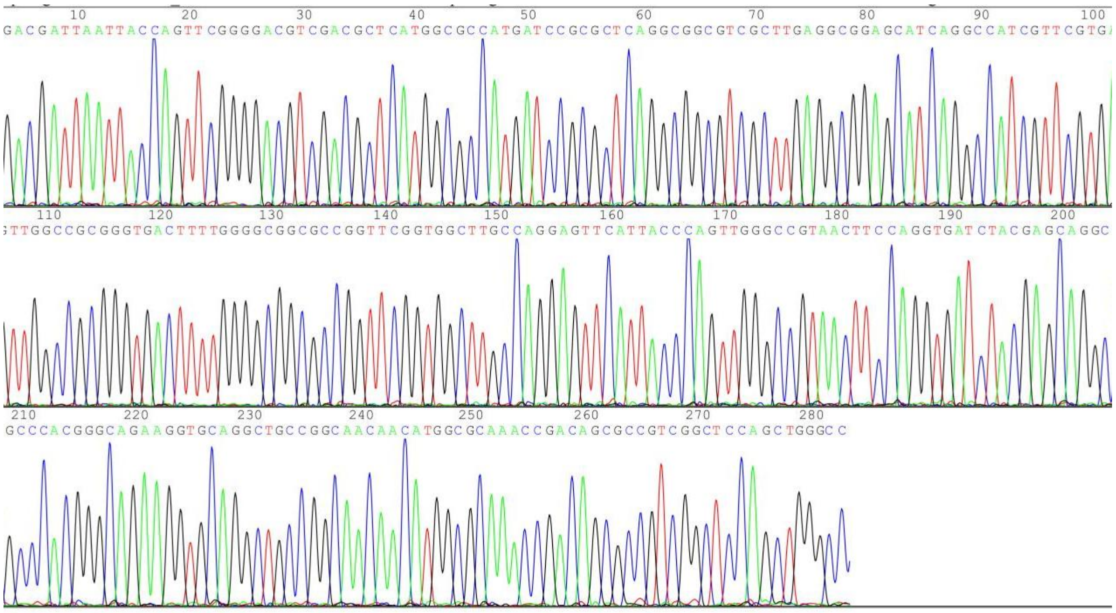

Supplement: Supplementary file 4 — Additional file 4: DNA sequencing. [file 11658_2024_585_MOESM4_ESM.pdf]
